# Reduced Introgression of Sex Chromosome Markers in the Mexican Howler Monkey (*Alouatta palliata* × *A. pigra*) Hybrid Zone

**DOI:** 10.1007/s10764-018-0056-4

**Published:** 2018-09-14

**Authors:** Liliana Cortés-Ortiz, Marcella D. Baiz, Javier Hermida-Lagunes, Francisco García-Orduña, Ariadna Rangel-Negrín, Dawn M. Kitchen, Thore J. Bergman, Pedro A. D. Dias, Domingo Canales-Espinosa

**Affiliations:** 1 Department of Ecology and Evolutionary Biology, University of Michigan, Ann Arbor, MI 48103 USA; 2 Instituto de Neuroetología, Universidad Veracruzana, Xalapa, Veracruz, Mexico; 3 Department of Anthropology, The Ohio State University, Columbus, OH 43210 USA; 4 Department of Psychology, University of Michigan, Ann Arbor, MI 48109 USA

**Keywords:** Haldane’s rule, Hybridization, Large X-effect, Reproductive isolation

## Abstract

Interspecific hybridization allows the introgression or movement of alleles from one genome to another. While some genomic regions freely exchange alleles during hybridization, loci associated with reproductive isolation do not intermix. In many model organisms, the X chromosome displays limited introgression compared to autosomes owing to the presence of multiple loci associated with hybrid sterility or inviability (the “large X-effect”). Similarly, if hybrids are produced, the heterogametic sex is usually inviable or sterile, a pattern known as Haldane’s rule. We analyzed the patterns of introgression of genetic markers located in the mitochondrial (control region) and nuclear (autosomal microsatellites and sex chromosome genes) genomes of two howler monkey species (*Alouatta palliata* and *A. pigra*) that form a natural hybrid zone in southern Mexico, to evaluate whether the large X-effect and Haldane’s rule affect the outcomes of hybridization between these sister species. To identify the level of admixture of each individual in the hybrid zone (*N* = 254) we analyzed individuals sampled outside the hybrid zone (109 *A. pigra* and 39 *A. palliata*) to determine allele frequencies of parental species and estimated a hybrid index based on nuclear markers. We then performed a cline analysis using individuals in the hybrid zone to determine patterns of introgression for each locus. Our analyses show that although the hybrid zone is bimodal (with no known F1 s and few recent generation hybrids) and quite narrow, there has been extensive introgression in both directions, and there is a large array of admixed individuals in the hybrid zone. Mitochondrial and most autosomal markers showed bidirectional introgression, but some had biased introgression toward one species or the other. All markers on the sex chromosomes and a few autosomal markers showed highly restricted introgression. This pattern is consistent with the hypothesis that the sex chromosomes make a disproportionate contribution to reproductive isolation, and our results broaden the taxonomic representation of these patterns across animal taxa.

**Electronic supplementary material:**

The online version of this article (10.1007/s10764-018-0056-4) contains supplementary material, which is available to authorized users.

## Introduction

Hybridization is the crossing of genetically distinct taxa that produces some viable offspring (Arnold 1997; Mallet 2005). In nature, this occurs in areas where two genetically divergent lineages meet and interbreed, creating regions referred to as hybrid zones (Barton and Hewitt 1985; Harrison 1990). In a hybrid zone, hybridization and recombination over many generations shuffle divergent genomes, and selection acts on the recombined genotypes, allowing the mixture of some genomic regions from the two parental lineages while preventing other regions from intermixing. This process produces individuals with distinct levels of admixture across their genome (Barton and Hewitt 1985; Harrison 1990; Hewitt 1988; Payseur 2010) and implies that genomes of hybridizing species are semipermeable to introgression (the movement of alleles from one gene pool into another through admixture; Parchman *et al.* 2013), with permeability depending on the characteristics of different genomic regions (Larson *et al.* 2013). It is expected that alleles that are advantageous in both parental genomes should easily spread through the hybrid zone, and neutral alleles should be freely exchanged between species. However, genomic regions that contribute to local adaptation or reproductive isolation should have restricted levels of introgression (Carneiro *et al.* 2013; Larson *et al.* 2013; Payseur 2010; Poelstra *et al.* 2014) and present relatively steep clines when plotted across a gradient of hybridization, either across a geographic transect or as a function of a hybrid index (i.e., the proportion of ancestry from each parental species) for admixed individuals (see Larson *et al.* 2013).

Two features are broadly documented when hybridization occurs among divergent lineages. First, individuals of the heterogametic sex (males in mammals) are more likely to be sterile or inviable than individuals of the homogametic sex. This pattern, known as Haldane’s rule (Haldane 1922), limits the introgression of loci in the mammalian Y chromosome (or the W chromosome of species with ZW sex chromosome systems). The second feature is the “large X-effect” (Coyne and Orr 1989; Masly and Presgraves 2007), or the observation that genes associated with reproductive barriers (e.g., hybrid sterility or inviability) are found at higher densities in the X chromosome (or the Z chromosome of species with ZW sex chromosome systems) than in autosomes. Consequently, studies of hybridizing taxa find that X-linked loci generally show reduced patterns of introgression in hybrid zones (Larson *et al.* 2014; Payseur and Nachman 2005; Tucker *et al.* 1992). However, owing to the relatively limited number of studies on hybrid zones across the taxonomic spectrum it is unclear whether this phenomenon is pervasive or particular to specific lineages.

The increased availability of genomic data has allowed the recognition of hybridization as a common phenomenon across all major primate lineages (see Arnold 2009; Cortés-Ortiz 2017 for reviews on the topic), with an estimated prevalence of natural hybridization in >10% of all primate species (Zinner *et al.* 2011). However, the quantification of genetic admixture in natural primate hybrid zones and the identification of social, ecological, and evolutionary processes that may be affecting interspecific gene flow across different genomic regions in primates are quite limited (Cortés-Ortiz 2017; Malukiewicz *et al.* 2014; Tung *et al.* 2008; Wall *et al.* 2016), despite the increased recognition of the importance of interspecific gene flow through hybridization during the evolution of several animal species (e.g., insects: Larson *et al.* 2014; Martin *et al.* 2013; birds: Poelstra *et al.* 2014; amphibians: Evans 2008; and mammals: Shurtliff 2013), including our own lineage (Ackermann *et al.* 2015; Sankararaman *et al.* 2014).

**Fig. 1:**
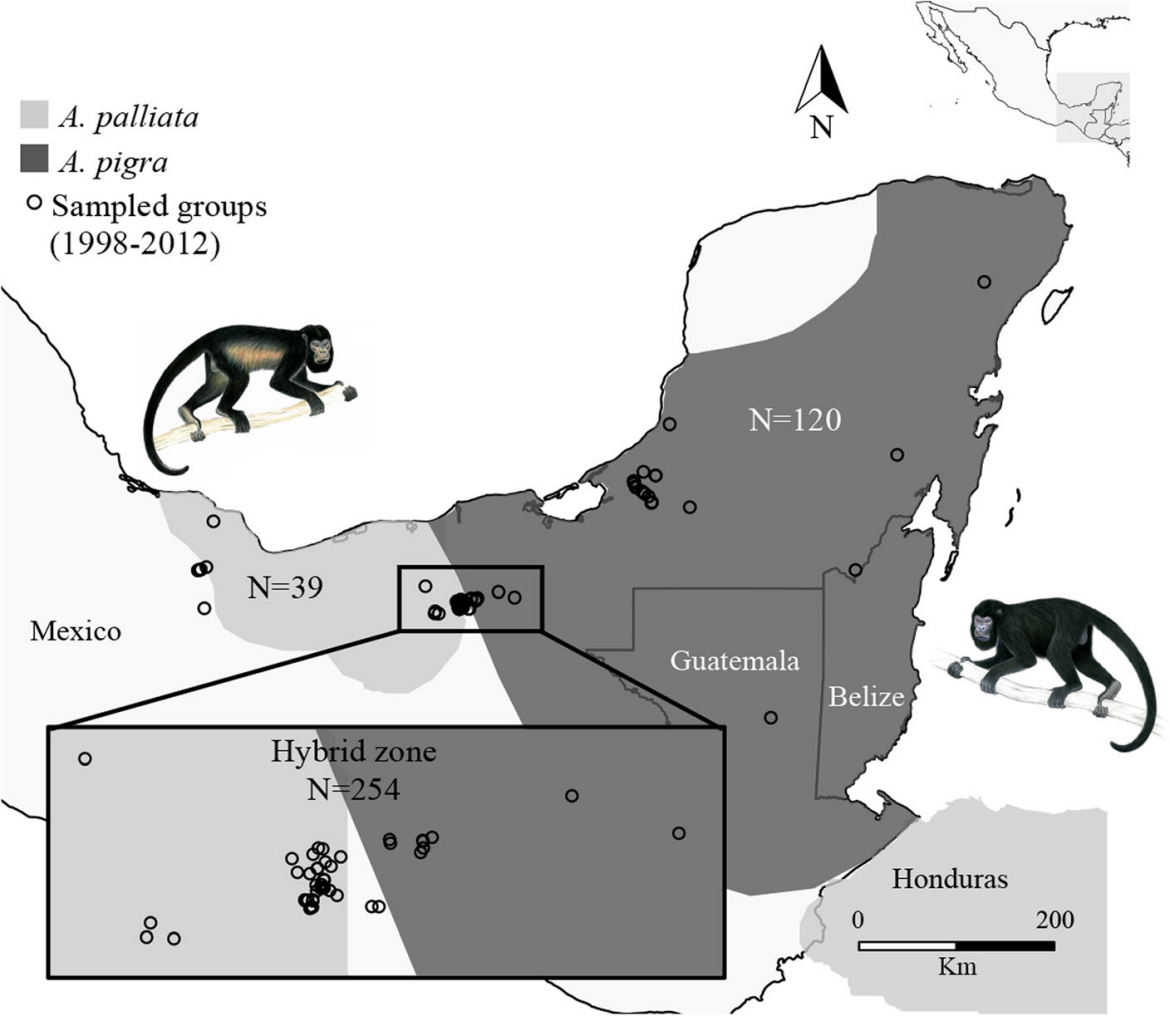
Distribution of *Alouatta palliata* (*light gray*) and *A. pigra* (*dark gray*) (based on IUCN 2008) showing location of sampled groups (*circles*) within and outside the hybrid zone. The inset is a close-up of groups in the hybrid zone. N = number of sampled individuals. Primate drawings by Stephen Nash.

The documented hybrid zone between mantled (*Alouatta palliata*) and black (*A. pigra*) howler monkeys in Mexico (Cortés-Ortiz *et al.* 2007) provides an opportunity to quantify genetic admixture and report the outcomes of hybridization and the consequent patterns of introgression between sister primate species that have not acquired complete reproductive isolation. *A. palliata* and *A. pigra* diverged ca. 3 MA (Cortés-Ortiz *et al.* 2003). These species are morphologically (Kelaita *et al.* 2011; Kelaita and Cortés-Ortiz 2013; Smith 1970), behaviorally (Ho *et al.* 2014), and genetically distinct (Cortés-Ortiz *et al.* 2003). They have different chromosome numbers (*A. palliata* 2n = 54/53 and *A. pigra* 2n = 58 due to several chromosomal rearrangements), and distinct multiple sex chromosome systems (X1X2Y in male *A. palliata* and X1X2Y1Y2 in male *A. pigra*) (Steinberg *et al.* 2008). These sex chromosome systems do not represent chromosome duplication events, but instead translocations of autosomal fragments into sex chromosomes that allow autosomes to pair to parts of the sex chromosomes during meiosis (e.g., Solari and Rahn 2005). The multiple sex chromosome systems of *A. palliata* and *A. pigra* have a common origin, which is distinct from the origin of the multiple sex chromosome systems in other *Alouatta* species (Steinberg *et al.* 2014). *A. palliata* has a broad distribution ranging from southern Mexico through Central America and the Pacific coast of northwestern South America (Fig. 1), with the Mexican population having a disjunct distribution to the rest of the species, likely owing to changes in climate after their expansion (Cortés-Ortiz 2017). *A. pigra* is restricted to the Peninsula of Yucatan in Mexico, Belize, and part of Guatemala (Fig. 1). Although the two species are mostly parapatric in Mexico, there is a small area of sympatry in the state of Tabasco (Horwich and Johnson 1986; Smith 1970), where hybridization has been documented (Cortés-Ortiz *et al.* 2007). It is likely that the area of overlap where these species hybridize is due to secondary contact as a consequence of range expansion after periods of isolation (Cortés-Ortiz *et al.* 2003; Ford 2006). The current hybrid zone in Mexico consists of a patchwork of groups of individuals of each parental species, mixed groups with individuals of both species or of parental and hybrid individuals, and groups composed entirely of hybrid individuals (Cortés-Ortiz *et al.* 2007, 2015). These groups co-occur in a narrow stretch covering an area of ≥67 km^2^ (Cortés-Ortiz *et al.* 2015). The narrowness of the hybrid zone is consistent with strong levels of reproductive isolation (e.g., selection against hybrids; Barton and Hewitt 1985) operating in this hybrid system.

Based on the distribution of mitochondrial and Y chromosome markers in a small number of hybrid individuals (i.e., adult individuals with only *Alouatta pigra* type mtDNA and males with only *A. pigra* type *SRY* gene), a previous study suggested Haldane’s rule is likely occurring in this hybrid system (Cortés-Ortiz *et al.* 2007). Considering the expected strong isolation between the parental species, together with the predictions of Haldane’s rule and the large X-effect on postzygotic isolation, we anticipate finding restricted introgression of the sex chromosomes. Here we examined and compared patterns of introgression for mitochondrial, autosomal, and sex chromosome (X-linked and Y-linked) markers within the *A. palliata* × *A. pigra* hybrid zone in Mexico to understand the genetic patterns of admixture and to test for the predicted differential patterns of introgression in autosomal vs. sex chromosome markers. We expand previous genetic analyses (Cortés-Ortiz *et al.* 2007) using a larger sample size that includes individuals in a broader geographic range within and outside the *A. palliata* × *A. pigra* hybrid zone, as well as an increased number of genetic markers to characterize the patterns of admixture in the hybrid zone.

## Methods

### Sampling

Between 1998 and 2012 we collected 413 blood samples from anesthetized wild howler monkey individuals of nonadmixed and admixed populations in Mexico and Guatemala (Cortés-Ortiz *et al.* 2007; Ho *et al.* 2014; Kelaita *et al.* 2011; Fig. 1, Electronic Supplementary Material [ESM] Table SI), following the capturing procedures described in Rodríguez-Luna and Cortés-Ortiz (1988) and blood collecting protocols detailed in Baiz and Cortés-Ortiz (2015). We preserved whole blood in lysis buffer (Seutin *et al.* 1991) at a 1:5 ratio and released individuals in the same location after they recovered from the effects of anesthesia. We recorded the GPS location (decimal coordinate data) for each sampled group; thus, each individual is associated with the GPS location where its group was sampled (Fig. 1). We also photographed individuals and marked some of them with ankle bracelets as part of behavioral studies (Ho *et al.* 2014). We genotyped (see later) all samples, but because we collected samples in the hybrid zone at different times and in a relatively small geographic area where individual dispersal is possible, we eliminated any samples collected during different sampling years that we suspected to belong to the same individual because of similarities in genotype and/or morphology. In this study we analyze 402 individuals from 78 distinct groups that include 109 *Alouatta pigra* outside the hybrid zone, 39 *A. palliata* outside the hybrid zone, and 254 individuals within the hybrid zone (135 that mostly resembled *A. palliata* in external morphology and 119 that mostly resembled *A. pigra*).

### Sequencing and Genotyping

We extracted genomic DNA from blood samples for all individuals using the QIAGEN DNeasy tissue kit (Qiagen Inc., Valencia, CA), following procedures described in Baiz and Cortés-Ortiz (2015). We used the polymerase chain reaction (PCR) to amplify the mitochondrial (mtDNA) control region and a panel of 29 microsatellite loci (including one on the X chromosome) for all samples (ESM Table SIII). Additionally, we amplified two X chromosome genes (*Zic3* and *Zfx*) for 137 individuals (25 males and 27 females from parental populations outside the hybrid zone and 85 individuals in the hybrid zone), and one Y chromosome marker (*SRY*) for 173 males (59 *Alouatta pigra* and 20 *A. palliata* outside the hybrid zone and 94 males from the hybrid zone) following procedures and conditions summarized in ESM Table SIII and described elsewhere (Cortés-Ortiz *et al.* 2007, 2010; Kelaita and Cortés-Ortiz 2013; Baiz and Cortés-Ortiz 2015; Perelman *et al.* 2011). We sent PCR products to the Sequencing Core Facilities of the University of Michigan (SCFUM) for Sanger sequencing in both directions of mtDNA, *SRY*, *Zic3*, and *Zfx* and for genotyping microsatellite loci as described in Baiz and Cortés-Ortiz (2015). The SCFUM ran all sequencing and genotyping on an ABI 3730xl DNA Analyzer. Mitochondrial and *SRY* sequences produced multiple unique haplotypes for each species and thus we considered them fully diagnostic to identify the maternal lineage of each individual and the paternal lineage of all males, respectively. The X chromosome genes *Zic3* and *Zfx* produced unique sequences for each species and thus we coded sequence data as genotypes for each individual (e.g., P1/P1, P1/P2 or P2/P2). We considered individuals to be homozygotes if we did not observe any double peaks in the chromatogram. We observed heterozygous genotypes only for four individuals, all of which were females. We combined these genotypes with the microsatellite data for introgression analyses.

### Admixture Analyses

To determine genetic admixture of individuals in the hybrid zone, we first observed microsatellite allele frequency differences between parental populations by generating heat maps using PopGenReport (Adamack and Gruber 2014) (ESM Fig. S1). We dropped five loci (D17S804, PEPC8, Apm4, Ab07, and Api07) from analyses because parental populations shared the most common allele at high frequency. These loci are not particularly informative for admixture analyses owing to uncertainty in inheritance for hybrids. In total we used genotypes of 24 microsatellite loci (23 autosomal and 1 on the X chromosome) to determine general levels of genetic admixture.

To define the level of genetic admixture of each individual in the hybrid zone we calculated a hybrid index using the program Introgress (Gompert and Buerkle 2009, 2010). The hybrid index is a maximum likelihood estimation of the proportion of an individual’s genome inherited from one of the parental species (Buerkle 2005), which is based on allele frequencies of codominant markers in the parental populations outside the hybrid zone. For each individual in the hybrid zone, the hybrid index represents the proportion of alleles inherited from *A. pigra* (where 1 = *A. pigra*, 0 = *A. palliata*, and 0.5 is a likely F1 hybrid). We also calculated interspecific heterozygosity, which is the proportion of loci that are heterozygous with an allele from each parental species (where 0 = either *A. pigra* or *A. palliata*, and 1 = likely F1 hybrid). To visualize the geographic distribution of genotypes in the hybrid zone, we plotted hybrid index scores for each sampling location using the package scatterpie on a map created with ggmap (Kahle and Wickham 2013) in R (R Core Team 2017).

### Differential Introgression Analyses

To analyze differential introgression, we first used the autosomal and X-chromosome microsatellite dataset for all individuals. To confirm the pattern of introgression for the X-chromosome observed in our single X-chromosome microsatellite, we conducted post hoc analyses with the genotype data of the two X-chromosome genes (*Zfx* and *Zic3*) using a reduced dataset. This dataset consisted of 26 *Alouatta palliata* and 26 *A. pigra* from outside of the hybrid zone and 85 individuals representing the gamut of admixed individuals from Tabasco. We used Introgress to analyze both the full and reduced datasets to measure genomic clines for each locus based on allele frequencies in the parental populations. For the microsatellite markers (present in both datasets), we compared results of the Introgress analyses to detect any effect of the reduced sample size. Locus-specific cline shape and individual hybrid index scores were consistent using loci represented in the full and reduced datasets (ESM Figs. S2a, b and S3). We report results using the full dataset for all microsatellite loci and only report results for *Zfx* and *Zic3* using the reduced dataset.

Briefly, we generated a neutral expectation of introgression using the permutation method implemented in the genomic.clines function of Introgress to conduct 10,000 neutral simulations based on observed allele frequencies. At each locus, this method uses multinomial regression to predict the probability of observing each possible genotype: 1) homozygous *Alouatta palliata* (Apa/Apa), 2) homozygous *A. pigra* (Api/Api), or 3) interspecific heterozygote (Apa/Api) given the hybrid index. This method cannot be used to analyze multiple types of markers (i.e., codominant and haploid) in the same analysis (Gompert and Buerkle 2010). Because we have only two haploid markers (*SRY* and mtDNA) and several markers are required to generate a neutral expectation, we calculated genomic clines only for our codominant dataset (i.e., microsatellites and two X-linked genes). We identified loci that deviate from the neutral expectation using the *P* value from the significance testing implemented with the genomic.clines function and determined the pattern of introgression for each locus by considering cline shape and whether homozygotes (either Apa/Apa or Api/Api), and heterozygotes (Apa/Api) were over- or underrepresented based on the neutral expectation. An excess or deficiency of homozygotes for the allele from one species (but not the other) is consistent with directional selection, while an excess or deficiency of heterozygotes is consistent with overdominance (i.e., heterozygote advantage) and underdominance (i.e., selection against heterozygotes and reduced introgression), respectively (Gompert and Buerkle 2009; Larson *et al.* 2013). We corrected for multiple comparisons using the false discovery rate (FDR) method to adjust *P*-values.

### Ethical Note

The University of Michigan Committee for the Use and Care of Animals (UCUCA) approved the protocols used for animal restraint and sample collection (permit # 09319). All samples were collected and exported with permission of the respective authorities in the countries of origin and legally imported into the United States (ESM Table SII). This research complies with the Guidelines of Best Practices for Field Primatology of the International Primatological Society.

The authors declare that they have no conflict of interest.

#### Data Availability

The datasets analyzed in this study are available from the corresponding author on reasonable request.

## Results

**Fig. 2:**
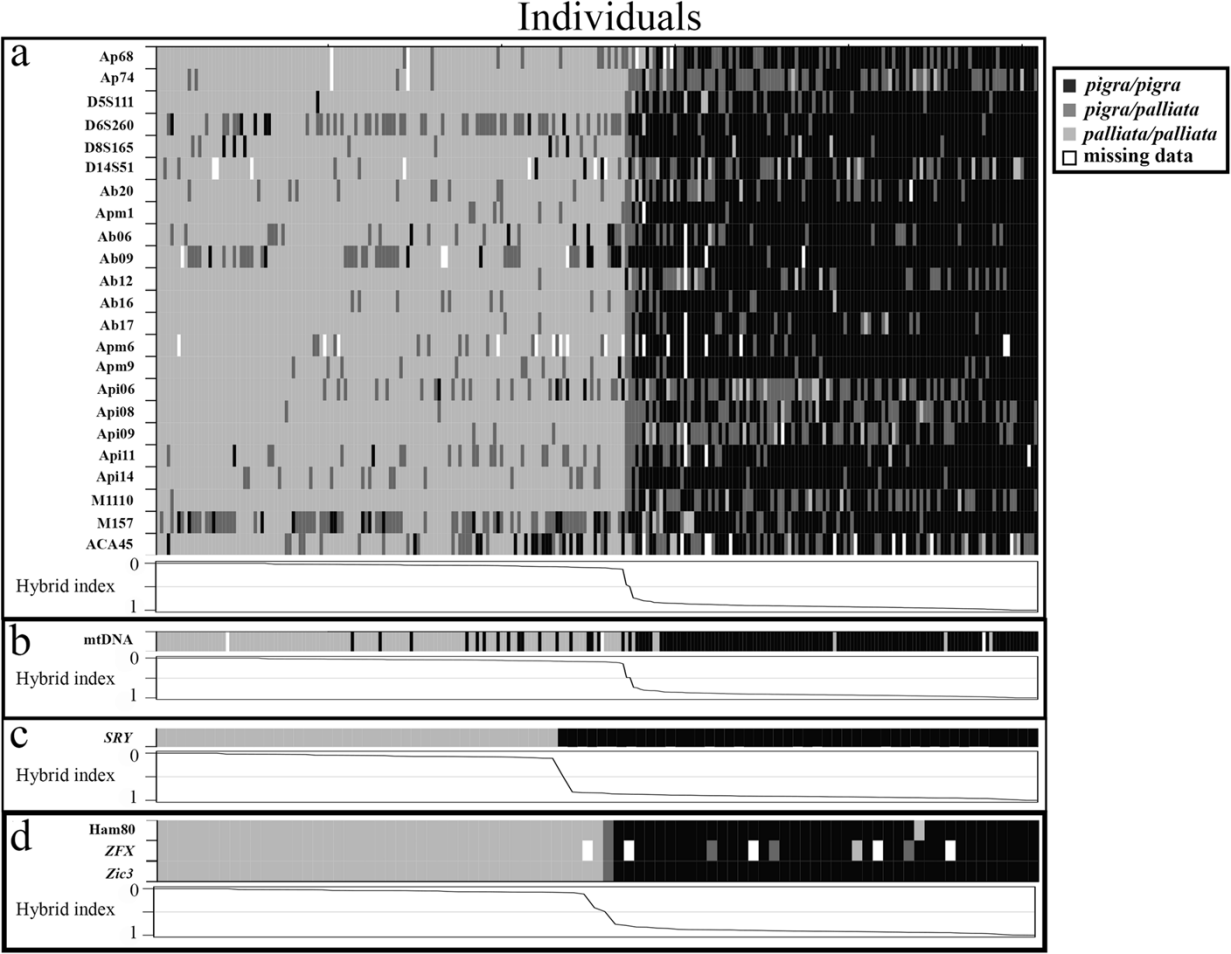
Genotypes of individuals in the *Alouatta palliata* × *A. pigra* hybrid zone in Tabasco, Mexico. Individuals are in columns arranged by their hybrid index as calculated using the codominant markers (0 = *A. palliata*, 1 = *A. pigra*) and markers are in rows. Black indicates homozygous *A. pigra* genotype, light gray is homozygous *A. palliata* genotype, dark gray is heterozygous genotype, and white is missing data. **a** Autosomal microsatellite genotypes (*N* = 254). **b** Mitochondrial DNA (*N* = 251). **c** Y-linked marker (*N* = 91). **d** X-linked markers (*N* = 85, i.e., reduced dataset).

Microsatellite genotypes for 254 individuals in the hybrid zone show a large array of admixed individuals (Fig. 2a), and only a few nonadmixed individuals for each species. When the specific identities of mitochondrial haplotypes of each admixed individual are compared with the corresponding hybrid index (Fig. 2b) it is apparent that some individuals whose genome comprises mostly *Alouatta pigra* alleles possess *A. palliata* mitochondrial haplotypes and vice versa, implying that females of both species have contributed to the production of hybrid offspring. In contrast, males whose genome is biased toward one species always share the *SRY* haplotype of that species (Fig. 2c), implying no introgression of the Y chromosome into the heterospecific genomic background. The X-chromosome markers (Fig. 2d) show no or reduced introgression. When introgression was detected in the X chromosome through analyses of the reduced dataset it was mostly *A. palliata* alleles into *A. pigra* backcrosses, but not the opposite (Fig. 2d). However, analyses of the full dataset for the Ham80 microsatellite locus detected four *A. palliata* backcrossed individuals harboring *A. pigra* alleles and seven *A. pigra* backcrossed individuals harboring *A. palliata* alleles (data not shown).

**Fig. 3:**
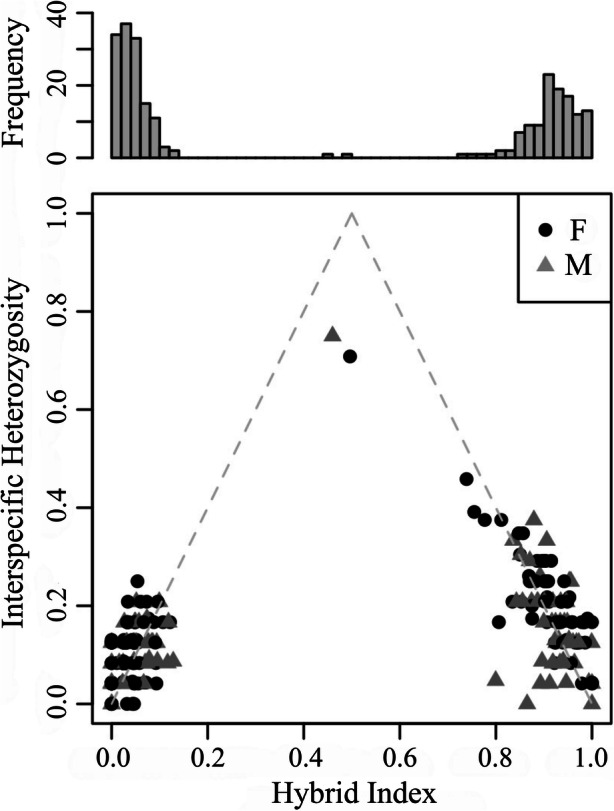
**a** Distribution of admixed genotypes in the *Alouatta palliata* × *A. pigra* hybrid zone in Tabasco, Mexico (*N* = 254). **b** Hybrid index plotted against interspecific heterozygosity for each individual in the hybrid zone. Hybrid index is represented as the proportion of alleles inherited from *A. pigra* (i.e., 0 = *A. palliata* and 1 = *A. pigra*) and interspecific heterozygosity is the proportion of loci that are heterozygous with an allele from each parental species. Individuals are coded by sex, where females (F) are represented as black dots and males (M) are gray triangles.

Hybrid index scores show a bimodal distribution (Fig. 3a), consistent with strong levels of reproductive isolation between the parental species (Jiggins and Mallet 2000). Admixture analyses revealed few recent-generation hybrids, but extensive introgression in the hybrid zone, and a bias for individuals backcrossed into *Alouatta pigra* to have higher variation in interspecific heterozygosity than individuals backcrossed into *A. palliata* (Fig. 3b). According to our hybrid index analyses 32 individuals in the hybrid zone could be characterized as nonadmixed *A. palliata* and 10 as nonadmixed *A. pigra* (i.e., hybrid index >0.99 or < 0.01). Nonetheless, three of these individuals had discordant mtDNA, and thus represent extremely backcrossed multigeneration hybrids. The majority of admixed individuals in the hybrid zone were highly backcrossed into either *A. palliata* (*N* = 103) or *A. pigra* (*N* = 107). Only two individuals (one male and one female) were identified as recent generation hybrids with high interspecific heterozygosity (0.75 and 0.71 respectively) and an intermediate hybrid index (0.46 and 0.50 respectively).

**Fig. 4:**
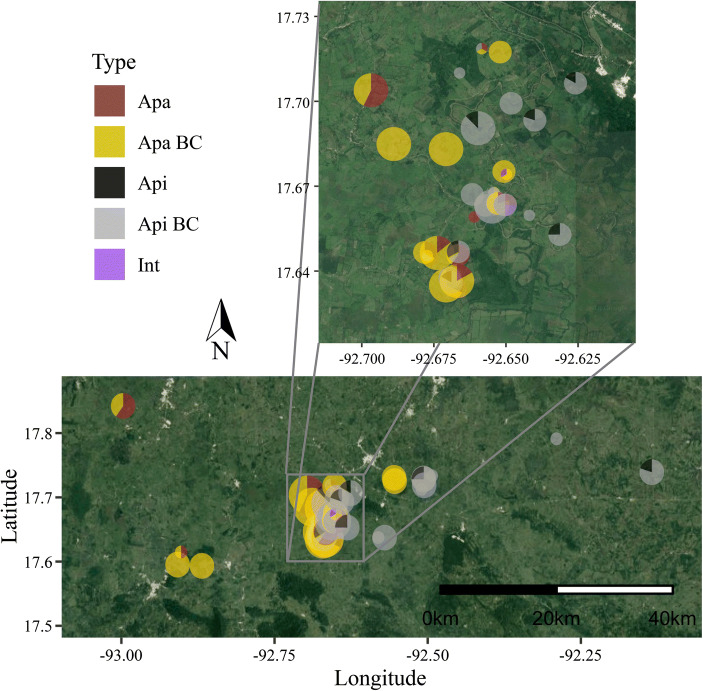
Geographic distribution of admixed and nonadmixed individuals in the *Alouatta palliata* (Apa) × *A. pigra* (Api) hybrid zone in Tabasco, Mexico (*N* = 254). The proportion of genotypes are represented for each sampling site, where Apa in brown is nonadmixed *A. palliata*, Apa BC in yellow represents backcrossed *A. palliata* hybrids, Api in black is nonadmixed *A. pigra*, Api BC in gray represents backcrossed *A. pigra*, and intermediate hybrids (Int) are in purple. The size of the pie is proportional to the number of individuals sampled at each site. The figure covers approximately the same area labeled as Hybrid Zone in Fig. 1. The zoomed-in area allows a clearer observation of the distribution of genotypes in each sampled group.

When considering the geographic distribution of admixed and nonadmixed individuals, we observe that individuals of both parental species and backcrosses are present throughout the hybrid zone (Fig. 4). Based on this distribution, the “center” of the hybrid zone seems to occur ca. –92.66°; most individuals west of this point are either nonadmixed or backcrossed *Alouatta palliata*, and most individuals east of this point are either nonadmixed or backcrossed *A. pigra*. This suggests that the region in the hybrid zone where nonadmixed individuals of both species might come into contact is quite narrow (<20 km). The two intermediate individuals also occur near this point. The female intermediate hybrid was sampled from a group (*N* = 5) containing one individual backcrossed into *A. pigra*, two *A. pigra* individuals, and one *A. palliata* individual. The male intermediate hybrid was sampled from a group containing two females backcrossed into *A. palliata*. Two other such mixed groups occurred in this central area, one group with three individuals, one *A. palliata*, one backcrossed into *A. palliata*, and one backcrossed into *A. pigra*, and the other group with only two individuals, one backcrossed into *A. palliata* and one backcrossed into *A. pigra*.

**Fig. 5:**
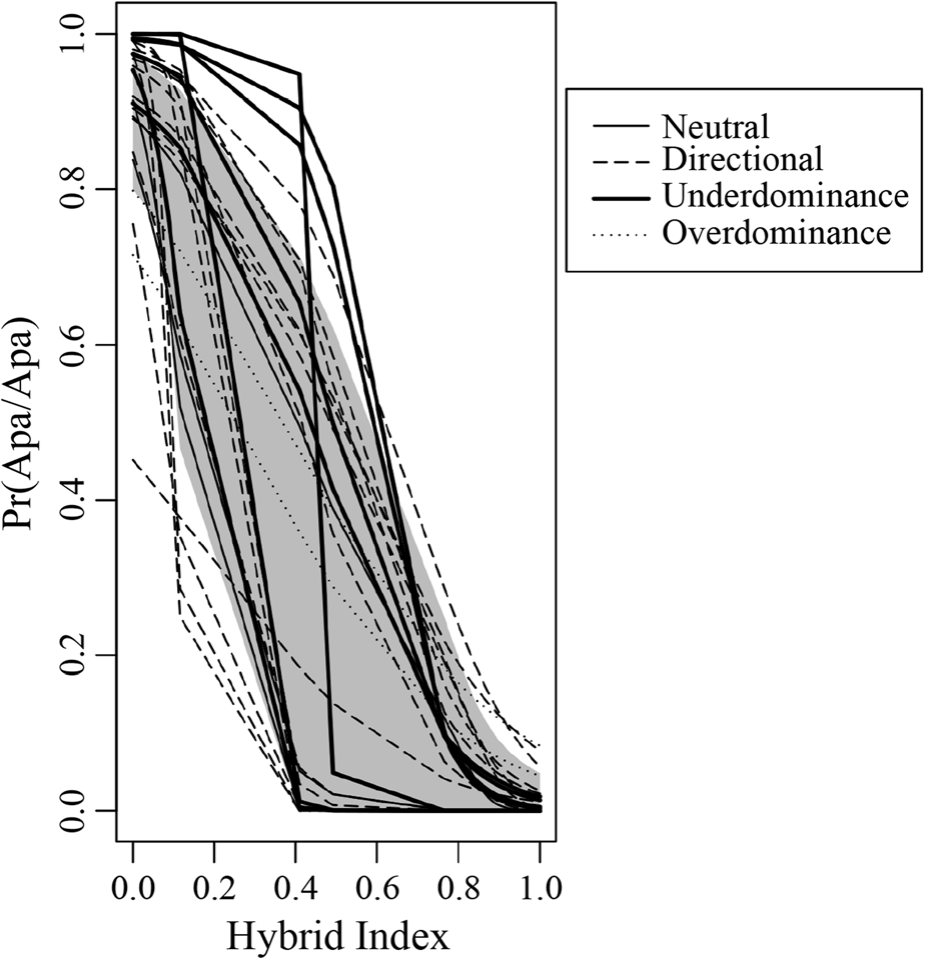
Genomic clines for 24 microsatellite (23 autosomal and one X-linked) markers and two X-linked genes, plotted as the probability of having a homozygous *Alouatta palliata* (Apa/Apa) genotype as a function of hybrid index, for individuals in the *A. palliata* × *A. pigra* hybrid zone in Tabasco, Mexico. Each line represents a cline for a single locus and the shaded gray area is the neutral expectation. Different line styles identify loci showing cline shape patterns consistent with particular types selection (thin solid line = neutral introgression; dashed line = directional selection; thick solid line = underdominance; dotted line = overdominance).

Introgress analyses showed that introgression varies among markers (Fig. 5). For some loci, alleles characteristic of one species are commonly present in individuals with an overall hybrid index biased to the other species, while other loci exhibited little or no evidence of introgression. In particular, the most common type of deviation was a shift in the cline center, with an excess of homozygotes for one parental class or the other, consistent with directional selection (*N* = 16), with 10 loci showing an excess of Apa/Apa genotypes (and/or a deficiency of Api/Api genotypes) and 6 loci showing an excess of Api/Api genotypes (and/or a deficiency of Apa/Apa genotypes) (ESM Fig. S2). Seven loci (the three X-linked markers and four autosomal microsatellites) showed a steep cline and a deficiency of heterozygotes indicating reduced introgression. Two loci had an excess of heterozygotes consistent with signatures of overdominance (i.e., where a heterozygous rather than a homozygous state in those loci may confer higher fitness to the hybrid), and two loci showed a pattern of introgression consistent with the neutral expectation.

## Discussion

Our results demonstrate high levels of genomic admixture and differential introgression across loci in the *Alouatta* hybrid zone in Mexico, but no or limited introgression of sex chromosome markers. The Y-linked locus (*SRY*) showed no introgression into the heterospecific genomic background, while two X-chromosome markers showed limited introgression (one bidirectional and the other unidirectional) and the third did not show signatures of introgression.

### Admixture Patterns in the Hybrid Zone

The majority of sampled individuals in the hybrid zone had a mosaic genome with a mix of alleles and haplotypes characteristic of both species. Although the majority of sampled individuals in the hybrid zone are admixed, we also found some nonadmixed individuals of both species, suggesting that there is ongoing influx of both parental forms into the hybrid zone. Only 3.9% of individuals in the hybrid zone were identified as nonadmixed *Alouatta pigra* and 12.6% were considered nonadmixed *A. palliata*. The majority of individuals (83.5%) were admixed to some extent. This contrasts with early analyses of this hybrid zone (Cortés-Ortiz *et al.* 2007) based on a limited sample size (*N* = 36 individuals in the hybrid zone) in which each parental species was calculated to comprise approximately one third of the individuals in this area, and only one third of individuals was considered to be admixed. This difference is likely due to our increased sampling of individuals in the hybrid zone (*N* = 254) and to the higher number of microsatellite markers used in this study, which allows more precise recognition of highly backcrossed individuals.

Most admixed individuals have an autosomal DNA composition highly biased toward one species or the other, likely the result of multiple generations of backcrossing, which creates a bimodal distribution of genetic admixture in the hybrid zone. Furthermore, despite the relatively large number of sampled individuals within the hybrid zone, we did not find any F1 hybrids. Only two individuals have microsatellite hybrid indexes suggestive of early generation hybrids (a male with HI = 0.46 and a female with HI = 0.50), and in both cases we have evidence indicating that neither are F1 individuals. The first of these individuals, a male, has concordant mtDNA and *SRY* haplotypes (both of *Alouatta pigra* type) despite having a similar share of microsatellite alleles from each parental species. In that case, it is likely that this individual is the product of a mating between a nonadmixed *A. pigra* male or a male highly backcrossed into *A. pigra* (there are no males with discordant *SRY* type when compared to their majority of genomic background), with a nonadmixed or highly backcrossed *A. palliata* female. The second individual, a female with HI = 0.50, was sampled multiple times, first in 2001 when she was infant and later (2007) as an adult. In 2001 we sampled and recorded the identity of her parents, an *A. palliata* female (HI = 0.00) and a male backcrossed into *A. pigra* (HI = 0.84). Therefore, we are confident that this female is not an F1 either. A similar distribution of genotypes has been observed in other natural primate hybrid zones, where a bimodal distribution is observed (e.g., Callithrix hybrid zone in Brazil; Malukiewicz *et al.* 2014) or a limited number of F1 individuals are found despite extensive and directed sampling in the area of contact (e.g., tarsier hybrid zone in Sulawesi; Merker *et al.* 2009).

F1 or early generation hybrids are often rare in hybrid zones (Beysard and Heckel 2014; Larson *et al.* 2014; Teeter *et al.* 2008; Turner *et al.* 2012). F1 individuals may be rare if they have reduced fitness (Barton and Hewitt 1985). However, if a few F1 individuals succeed in siring offspring and backcrossing occurs, recombination may facilitate the appropriate combinations of genes, allowing backcrossed individuals to attain similar levels of fitness as parental individuals. Reduction in fitness of hybrids could partially explain the lack of observation of F1 individuals in our sample. However, in populations where hybridization has been ongoing for several generations and dispersal of the parental forms is limited, the increased density of admixed vs. nonadmixed individuals may also contribute to the reduced probability of producing F1 s, even if there is no drastic reduction in hybrid fitness (e.g., Larson *et al.* 2013; Tung *et al.* 2008). In fact, the *A. palliata* × *A. pigra* hybrid zone in Mexico is dominated by a large number of multigenerational backcrosses (see Fig. 3) that are more likely to find each other than nonadmixed individuals to reproduce (Dias *et al.* 2013), making it less likely to find F1 hybrids (Harrison and Larson 2016), although they may still occasionally occur. In contrast, a recent study of two Brazilian hybrid zones between black-and-gold (*A. caraya*) and brown (*A. g. clamitans*) howler monkeys, in which there is also a large number of backcrossed individuals, suggests the presence of six male and three female F1 s (Mourthe *et al.* 2018). Whether differences in demography, in levels of reproductive isolation between hybridizing taxa, or methodological disparities are responsible for the different proportions of F1 hybrids observed in the Mexican and Brazilian howler monkey hybrid zones awaits further study.

The combination of uni- and biparentally inherited markers we used in this study allows us evaluate possible asymmetry in hybridization. The mtDNA marker shows an approximately equal number of admixed individuals with *Alouatta palliata* and *A. pigra* haplotypes. However, analyzing the distribution of mtDNA haplotypes across the microsatellite hybrid index (Fig. 2b), more admixed individuals that are genetically closer to *A. palliata* (i.e., 0.00 < HI <0.30) have heterospecific mtDNA haplotypes (i.e., *A. pigra* haplotypes) than the opposite (14.5% vs. 5.6%, respectively). So, although backcrossing is occurring in both directions, there seems to be a bias with more *A. pigra* females (or admixed females with *A. pigra* maternal lineages) successfully producing hybrid offspring than *A. palliata* females. Whether this may be due to differences in social behavior (Cortés-Ortiz *et al.* 2015; Ho *et al.* 2014) or to genetic mechanisms also remains to be studied.

The distribution of the Y chromosome marker among admixed individuals presents a contrasting pattern, in which no admixed males show *SRY* haplotypes that are discordant with the majority of their genetic background. This observation is consistent with Haldane’s rule, where hybrids of the heterogametic sex are often inviable or infertile (Haldane 1922). If males were produced and were fertile in the F1, we then would expect that during backcrossing their Y chromosome would be passed to later generation hybrids in a similar fashion as we observe for the mtDNA. Instead, of the 91 males for which *SRY* was genotyped (42 admixed males that are genetically closer to *Alouatta palliata* and 49 that are genetically closer to *A. pigra*), none had *SRY* haplotypes discordant to the majority of their nuclear genomic background. This can be explained if during the first generation of hybridization (i.e., during the crossing of nonadmixed individuals of each of the parental species) males either are not sired or are infertile, and only after several backcrossing episodes (when F1 or backcrossed individuals mate between them or with nonadmixed individuals), and with the shuffling of genomes by recombination, appropriate genetic combinations allow the production of viable or fertile males. The presence of males across the entire genotype spectrum in the hybrid zone shows that this process is occurring in both directions. The observation of a hybrid male with a 0.46 microsatellite HI does not support the hypothesis that male hybrids in early generations are not viable (see also the foregoing explanation for the possible lack of F1 s due to demographic issues). Instead, it seems likely that, if produced, F1 (and other early generation) males may be infertile. In support of this hypothesis is the fact that the male with intermediate HI lived as an adult in a small group with two admixed females for ≥7 years, with no evidence of producing offspring during this period. Contrary to what is observed in the Mexican hybrid zone, the Brazilian howler monkey hybrid zones have admixed males with *SRY* haplotypes discordant to the identity of their autosomal alleles (Mourthe *et al.* 2018). This suggests that despite a deeper divergence between *A. caraya* and *A. clamitans* (estimated at ca. 5.1 MA; Cortés-Ortiz *et al.* 2003) the levels of reproductive isolation may be stronger in the *A. palliata* × *A. pigra* hybrid zone. Studies of other primate hybrid zones (e.g., macaques; Bonhomme *et al.* 2009; Tosi *et al.* 2002; baboons: Charpentier *et al.* 2008; tarsiers: Merker *et al.* 2009) have observed unidirectional bias in the introgression of Y chromosome markers, which has been attributed to a bias in dispersal or to behavioral differences between species, but no other studies have yet reported a complete lack of Y chromosome introgression as observed in this study. Further behavioral and genetic analyses of admixed males will be required to elucidate the processes and mechanisms that underlie this pattern in the Mexican hybrid zone.

### Width of Hybrid Zone and Geographic Distribution of Hybrids

In this study we observed that *Alouatta palliata*–like, *A. pigra*–like, and mixed groups are interspersed in a narrow area no more than 20 km wide. Beyond this narrow stretch, only individuals that morphologically resemble *A. palliata* (to the west) and *A. pigra* (to the east) exist. The presence of a narrow hybrid zone is commonly considered a result of secondary contact between formerly allopatric populations, in which hybrids have reduced fitness and gene flow is limited (Barton and Hewitt 1985). There is evidence that the *A. palliata* × *A. pigra* hybrid zone in Mexico is the result of secondary contact after periods of isolation (Cortés-Ortiz *et al.* 2003; Ford 2006) when the two species may have developed some extent of reproductive isolation due to drift or to adaptation to different environments. Narrow hybrid zones like the one observed in this system have been observed among a broad range of animals including crickets (e.g., Larson *et al.* 2013), frogs (Blackwell and Bull 1978), birds (e.g., Miller *et al.* 2014), and mammals (e.g., voles: Beysard and Heckel 2014; rabbits: Carneiro *et al.* 2013). In voles (genus *Micrutus*) a narrow hybrid zone occurs only between lineages that had an older divergence time, and in which signatures of selection against hybrid males are apparent (Beysard and Heckel 2014). The 3 MA of divergence between *A. palliata* and *A. pigra,* as well as the observed patterns of admixture consistent with strong levels of reproductive isolation, may explain the presence of a narrow hybrid zone between these species.

### Differential Introgression of Autosomal and Sex Chromosome Markers

The results of the genomic cline analysis showed great variation in the patterns of introgression among microsatellite markers, implying genomic heterogeneity of introgression. Some loci showed elevated introgression into the heterospecific genomic background, while other loci presented very limited introgression. Most autosomal microsatellite loci showed clines that are consistent with directional selection, with a large proportion of these loci having an excess of *Alouatta palliata* alleles, and several having an excess of *A. pigra* alleles. Although we cannot determine the actual location of these loci in the *Alouatta* genome, it is unlikely that all these markers are physically linked given that there is no consistent linkage disequilibrium between markers in populations of either parental species (Baiz and Cortés-Ortiz 2015). Therefore, the biased patterns of introgression observed may imply that several regions are adaptively introgressing. Nonetheless, given that our dataset is comprised of microsatellite loci of unknown location in the genome and unknown association with particular genes or functions, this hypothesis needs to be tested with future genomic data.

All X-linked markers tested (*N* = 3) and four presumably autosomal microsatellite loci showed evidence of restricted introgression. This suggests reproductive isolation may have both X chromosomal and autosomal components. It is widely accepted that the X chromosome plays a large role in speciation, particularly in postzygotic isolation (Coyne and Orr 1989; Masly and Presgraves 2007). Our observation of restricted introgression for X-linked markers relative to autosomal markers is consistent with other studies in animal systems that found reduced introgression of the X (or Z) chromosome (Carling and Brumfield 2008; Larson *et al.* 2014; Tucker *et al.* 1992), and where evidence for a large X-effect has been demonstrated (Masly and Presgraves 2007; Turner and Harr 2014). Although our dataset is a modest representation of the genome, we suspect that a large X-effect is operating in this system and may be involved in preventing the fusion of the two parental species. Similar analyses in other primate hybrid systems that show strong bimodal distributions of admixed individuals (e.g., Malukiewicz *et al.* 2014; Merker *et al.* 2009) are needed to understand if this phenomenon is pervasive across primates.

Although we could not explicitly test for nonneutral introgression in mtDNA or *SRY* using genomic cline analyses (see Methods), we examined the distribution of their haplotypes across the gradient of admixture for hybrids. Our results show that there has been some exchange mtDNA haplotypes of both species in hybrids (Fig. 2b), suggesting that mtDNA is selectively neutral in the hybrid zone. However, for *SRY*, all backcrossed hybrid males have the *SRY* haplotype that matches the parental species with which they share most of their genetic ancestry, showing that introgression of this locus is restricted, and suggesting a possible role of the Y chromosome in reproductive isolation (i.e., selection against Y chromosomes from one species on the predominant genetic background of the other in hybrid males). Restricted introgression of Y-linked markers could also occur in the absence of selection if the migration of males were more limited than the migration of females across the hybrid zone. Behavioral studies report bisexual dispersal in both *Alouatta palliata* and *A. pigra* populations (Brockett *et al.* 2000; Clarke and Glander 2008; Glander 1992; Horwich *et al.* 2000; Baiz and Cortés-Ortiz 2015; Van Belle *et al.* 2008), but patterns of dispersal have not been systematically evaluated in the hybrid zone. If bisexual dispersal also occurs in the hybrid population, then it is more plausible that the lack of introgression of the Y chromosome marker is due to selection against hybrids. Long-term behavioral studies in the hybrid zone will be needed to test the role of male and female dispersal and interspecific interactions in shaping the hybridization process in this system.

Our results demonstrate differential introgression among nuclear markers in the *Alouatta palliata* × *A. pigra* hybrid zone, including restricted introgression of sex chromosomes. Although the markers used in this study do not allow us to determine specific genomic regions that have patterns of introgression consistent with reproductive isolation, our results reveal that isolation mechanisms are already present (and likely strong) between *A. palliata* and *A. pigra*. The broad understanding of differential introgression acquired through this study leaves us in a good position to analyze differential introgression using a large set of genomic markers throughout the genome to identify particular genomic regions and understand mechanisms underlying reproductive isolation between these species.

## Electronic supplementary material

ESM 1 (PDF 924 KB)

ESM 2 (PDF 180 KB)
