# Supplementary material for: Reduced Introgression of Sex Chromosome Markers in the Mexican Howler Monkey (Alouatta palliata × A. pigra) Hybrid Zone
Source: Int J Primatol. 2018 Sep 14;40(1):114–31. doi: 10.1007/s10764-018-0056-4 (PMC6394575; doi:10.1007/s10764-018-0056-4)
Supplement: Supplementary file 1 — (PDF 924 KB) [file 10764_2018_56_MOESM1_ESM.pdf]

Figure S1

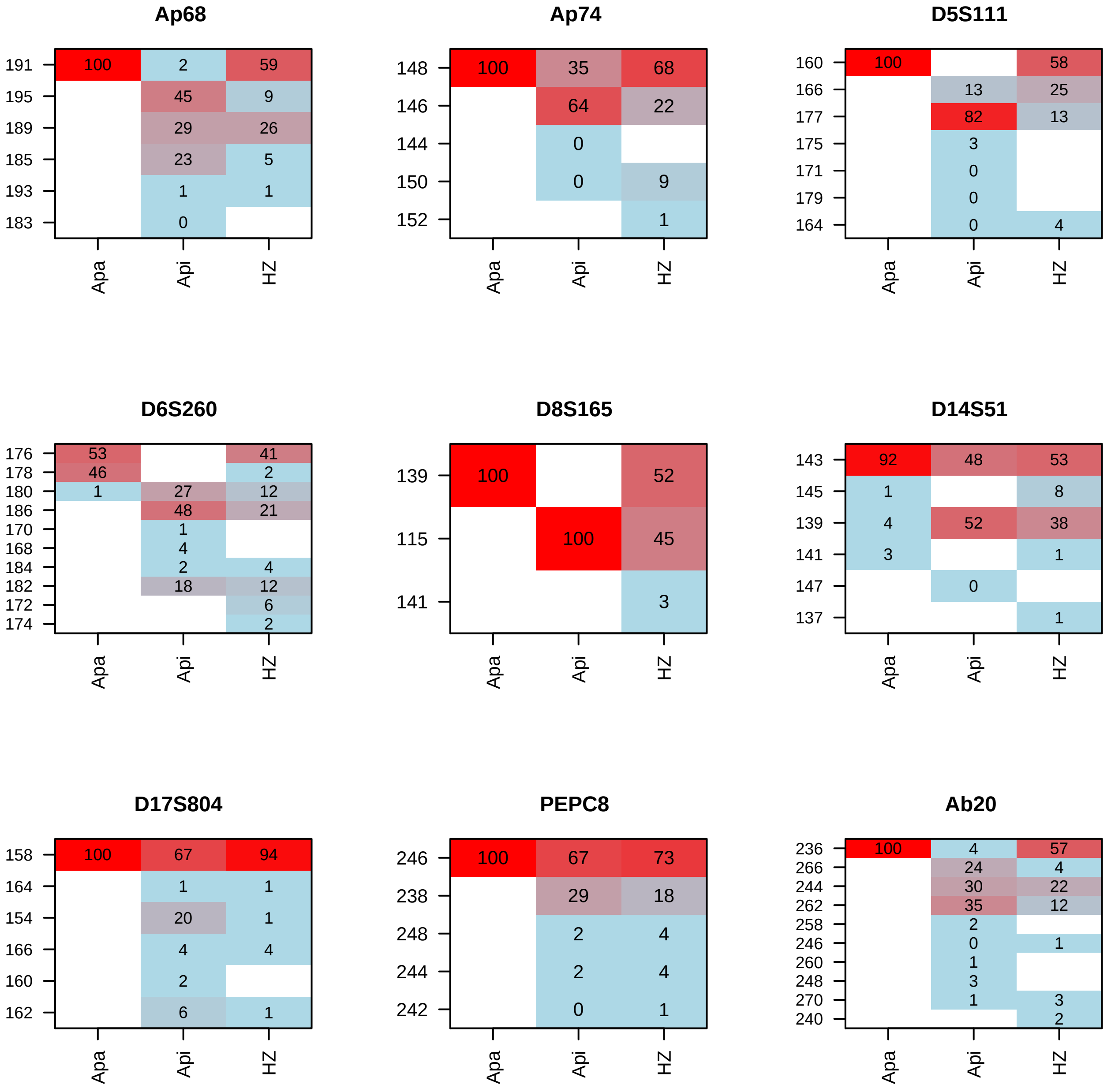
Ap68
191 195 189 185 193 183
100 2 59
45 9
29 26
23 5
1 1
0
Apa Api HZ
Ap74
148 146 144 150 152
100 35 68
64 22
0
0 9
1
Apa Api HZ
D5S111
160 166 177 175 171 179 164
100 58
13 25
82 13
3
0
0
0 4
Apa Api HZ
D6S260
176 178 180 186 170 168 184 182 172 174
53 41
46 2
1 27 12
48 21
1
4
2 4
18 12
6
2
Apa Api HZ
D8S165
139 115 141
100 52
100 45
3
Apa Api HZ
D14S51
143 145 139 141 147 137
92 48 53
1 8
4 52 38
3 1
0
1
Apa Api HZ
D17S804
158 164 154 166 160 162
100 67 94
1 1
20 1
4 4
2
6 1
Apa Api HZ
PEPC8
246 238 248 244 242
100 67 73
29 18
2 4
2 4
0 1
Apa Api HZ
Ab20
236 266 244 262 258 246 260 248 270 240
100 4 57
24 4
30 22
35 12
2
0 1
1
3
1 3
2
Apa Api HZ

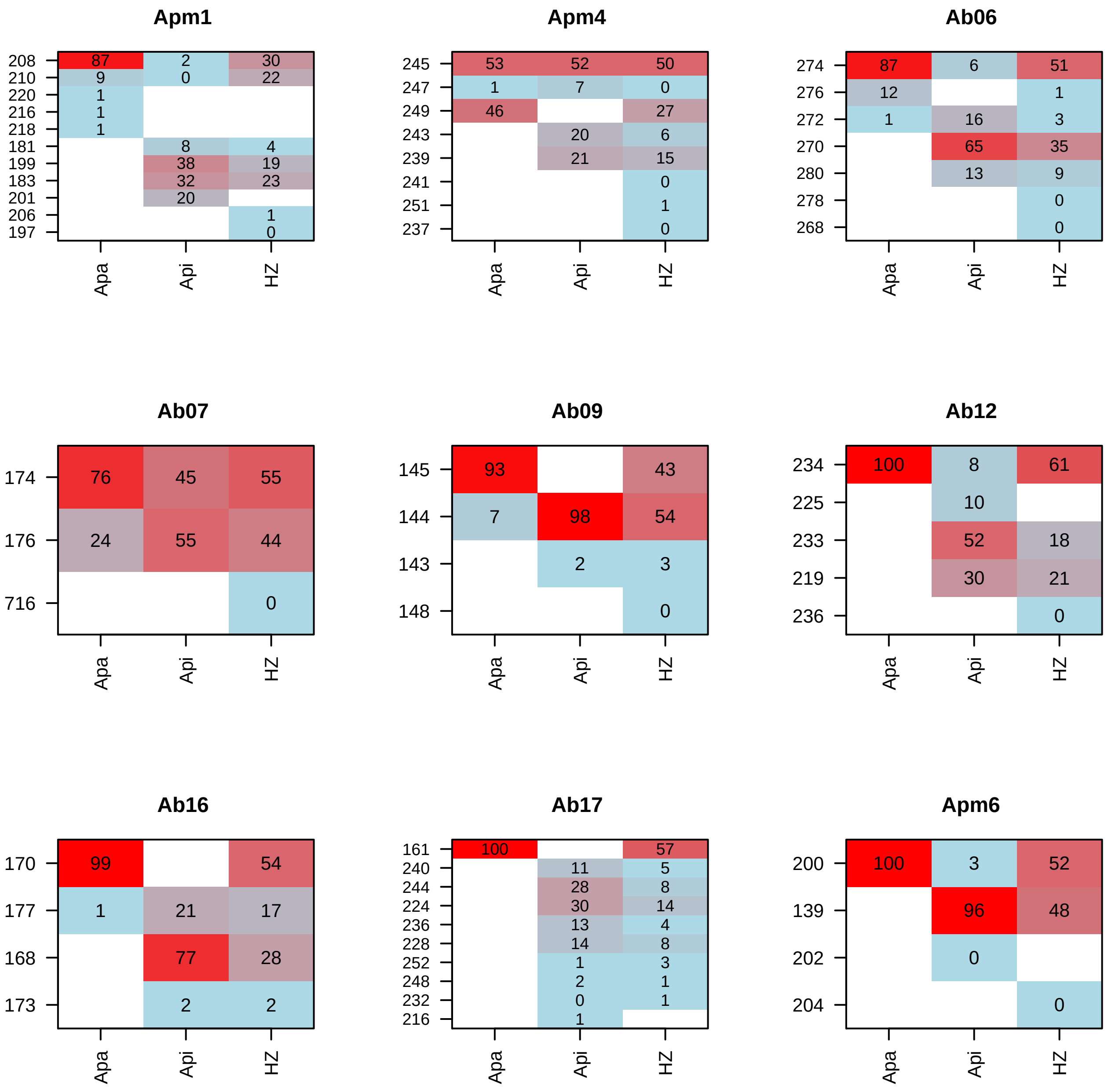
Apm1
208 210 220 216 218 181 199 183 201 206 197
87 2 30
9 0 22
1
1
1
8 4
38 19
32 23
20
1
0
Apa Api HZ
Apm4
245 247 249 243 239 241 251 237
53 52 50
1 7 0
46 27
20 6
21 15
0
1
0
Apa Api HZ
Ab06
274 276 272 270 280 278 268
87 6 51
12 1
1 16 3
65 35
13 9
0
0
Apa Api HZ
Ab07
174 176 716
76 45 55
24 55 44
0
Apa Api HZ
Ab09
145 144 143 148
93 43
7 98 54
2 3
0
Apa Api HZ
Ab12
234 225 233 219 236
100 8 61
10
52 18
30 21
0
Apa Api HZ
Ab16
170 177 168 173
99 54
1 21 17
77 28
2 2
Apa Api HZ
Ab17
161 240 244 224 236 228 252 248 232 216
100 57
11 5
28 8
30 14
13 4
14 8
1 3
2 1
0 1
1
Apa Api HZ
Apm6
200 139 202 204
100 3 52
96 48
0
0
Apa Api HZ

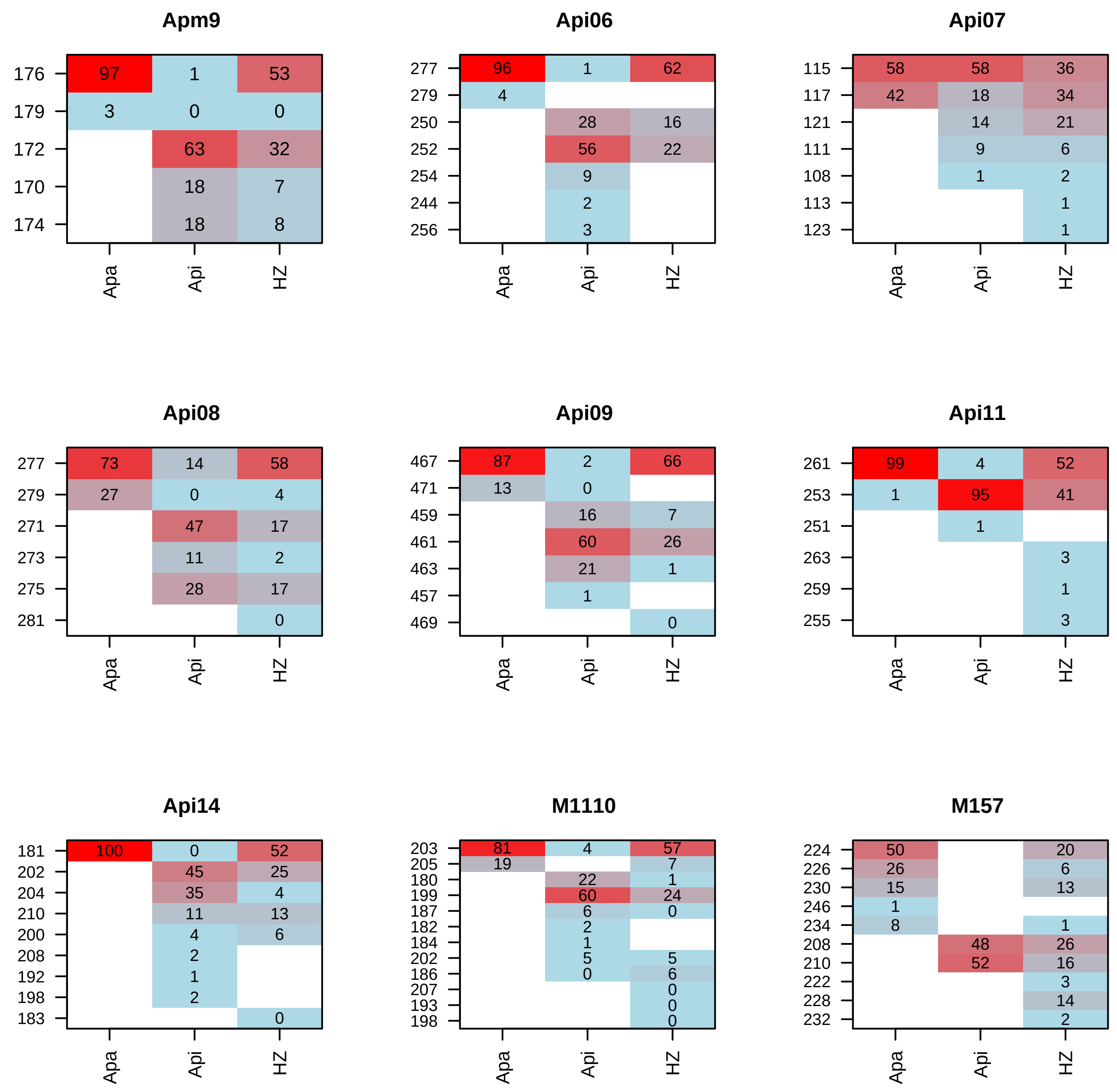

Apm9
176 97 1 53
179 3 0 0
172 63 32
170 18 7
174 18 8
Apa Api HZ
Api06
277 96 1 62
279 4
250 28 16
252 56 22
254 9
244 2
256 3
Apa Api HZ
Api07
115 58 58 36
117 42 18 34
121 14 21
111 9 6
108 1 2
113 1
123 1
Apa Api HZ
Api08
277 73 14 58
279 27 0 4
271 47 17
273 11 2
275 28 17
281 0
Apa Api HZ
Api09
467 87 2 66
471 13 0
459 16 7
461 60 26
463 21 1
457 1
469 0
Apa Api HZ
Api11
261 99 4 52
253 1 95 41
251 1
263 3
259 1
255 3
Apa Api HZ
Api14
181 100 0 52
202 45 25
204 35 4
210 11 13
200 4 6
208 2
192 1
198 2
183 0
Apa Api HZ
M1110
203 81 4 57
205 19 7
180 22 1
199 60 24
187 6 0
182 2
184 1
202 5 5
186 0 6
207 0
193 0
198 0
Apa Api HZ
M157
224 50 20
226 26 6
230 15 13
246 1
234 8 1
208 48 26
210 52 16
222 3
228 14
232 2
Apa Api HZ

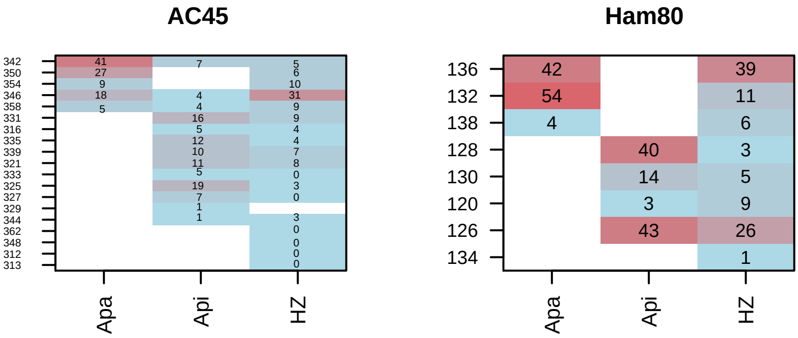

Fig. S1 Per locus heat maps showing the frequencies of alleles (presented as the percentages) by population. For each locus, alleles are denoted by size on the *y*-axis; populations are on the *x*-axis (Apa = *A. palliata* sampled outside the hybrid zone, Api = *A. pigra* sampled outside the hybrid zone, and HZ = individuals sampled in the hybrid zone). In the plot area, warmer colors indicate a higher percentage of individuals with a given genotype. Percentages ≤0.5 were rounded down to 0. We dropped from analyses loci where the most common allele in Apa was also the most common allele in Api. This included D17S804, PEPC8, Apm4, and Api07. We also dropped Ab07 because although the most common allele was different between populations, alternative alleles were present at nearly equal frequency in Api and the locus was not near fixation in Apa.

Figure S2A

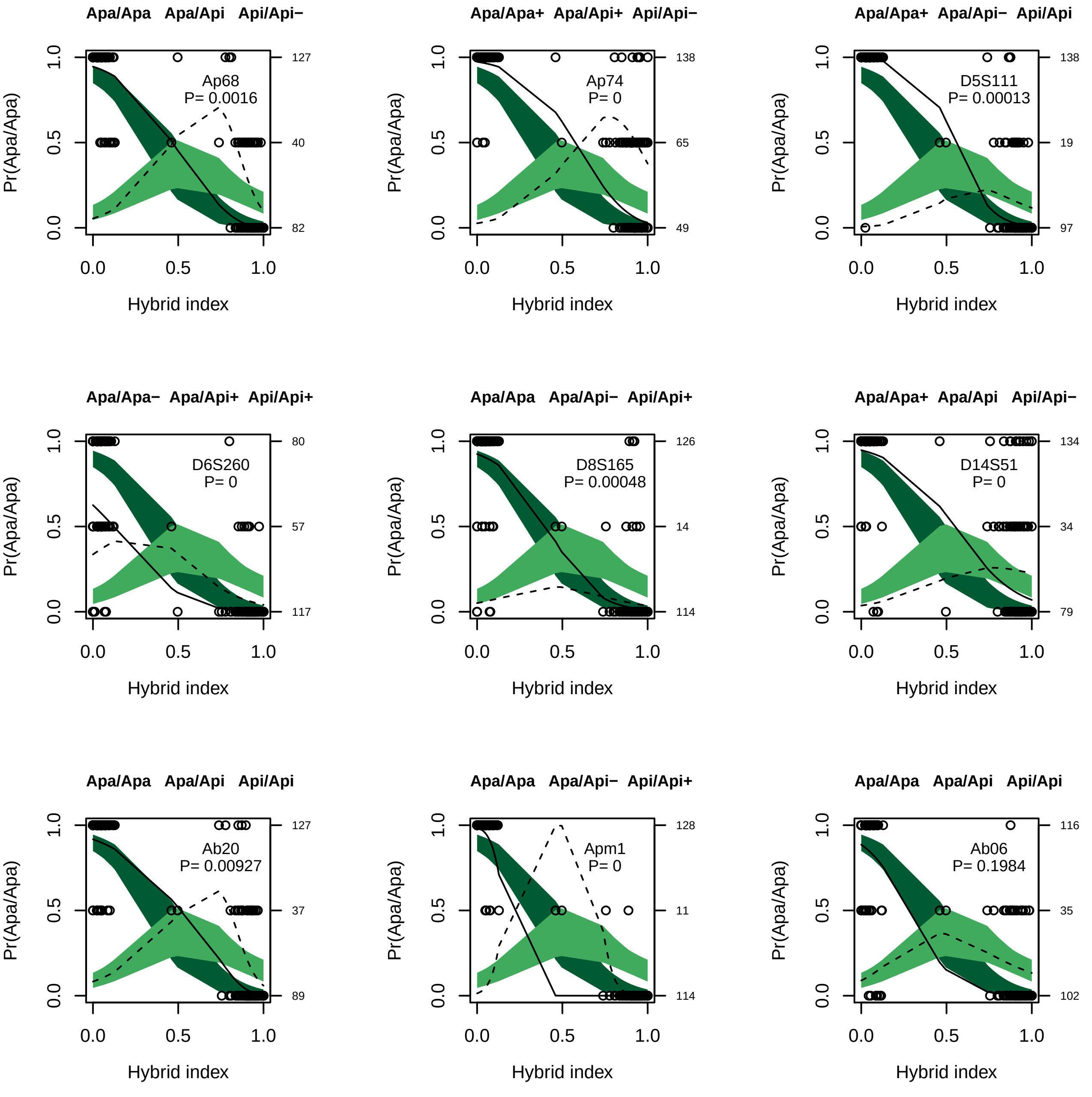
Apa/Apa Apa/Api Api/Api−
Ap68
P= 0.0016
127
40
82
Apa/Apa+ Apa/Api+ Api/Api−
Ap74
P= 0
138
65
49
Apa/Apa+ Apa/Api− Api/Api
D5S111
P= 0.00013
138
19
97
Apa/Apa− Apa/Api+ Api/Api+
D6S260
P= 0
80
57
117
Apa/Apa Apa/Api− Api/Api+
D8S165
P= 0.00048
126
14
114
Apa/Apa+ Apa/Api Api/Api−
D14S51
P= 0
134
34
79
Apa/Apa Apa/Api Api/Api
Ab20
P= 0.00927
127
37
89
Apa/Apa Apa/Api− Api/Api+
Apm1
P= 0
128
11
114
Apa/Apa Apa/Api Api/Api
Ab06
P= 0.1984
116
35
102
Pr(Apa/Apa)
Hybrid index
0.0
0.5
1.0

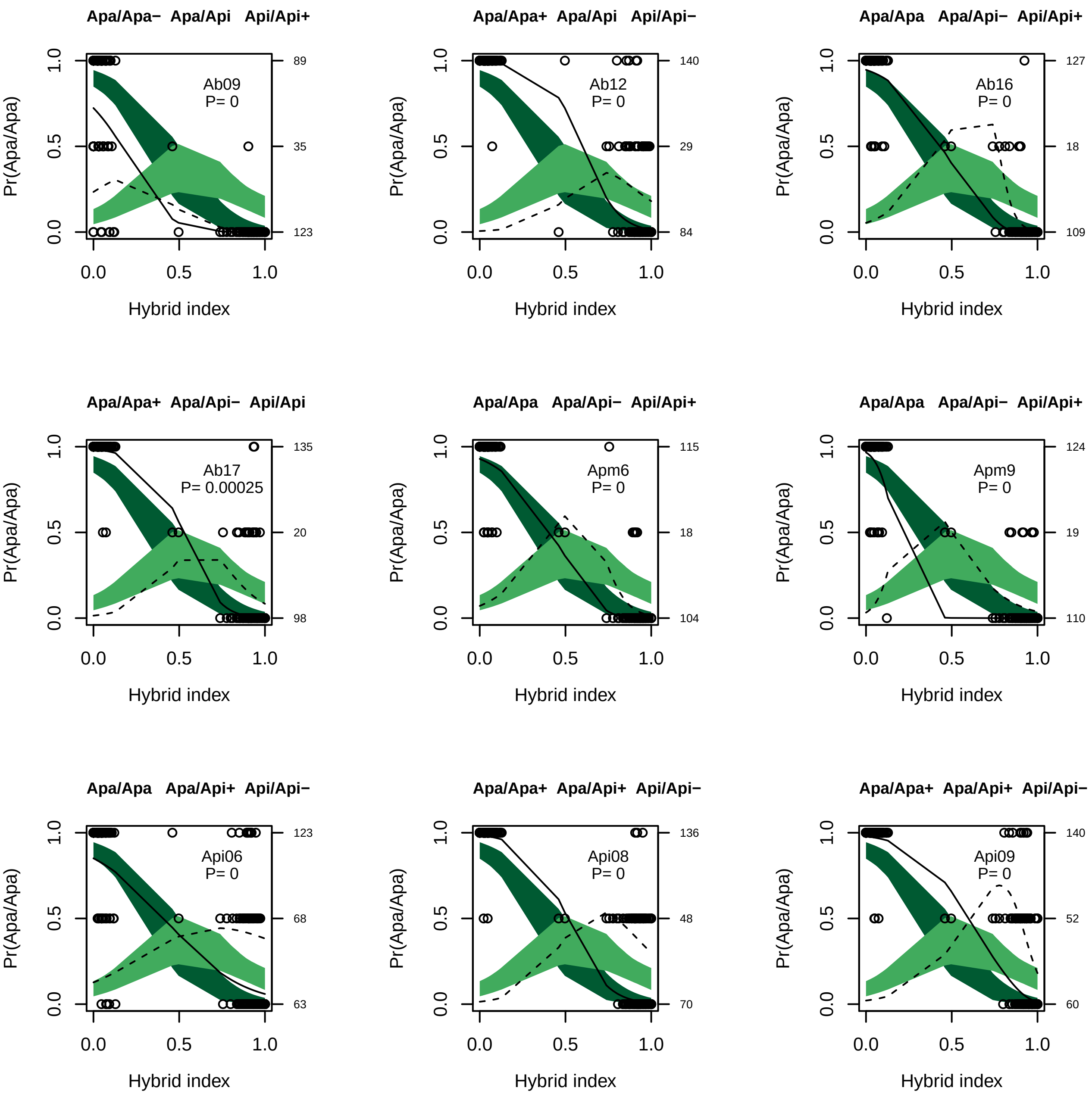

Apa/Apa− Apa/Api Api/Api+
Ab09
P= 0
89
35
123
Apa/Apa+ Apa/Api Api/Api−
Ab12
P= 0
140
29
84
Apa/Apa Apa/Api− Api/Api+
Ab16
P= 0
127
18
109
Apa/Apa+ Apa/Api− Api/Api
Ab17
P= 0.00025
135
20
98
Apa/Apa Apa/Api− Api/Api+
Apm6
P= 0
115
18
104
Apa/Apa Apa/Api− Api/Api+
Apm9
P= 0
124
19
110
Apa/Apa Apa/Api+ Api/Api−
Api06
P= 0
123
68
63
Apa/Apa+ Apa/Api+ Api/Api−
Api08
P= 0
136
48
70
Apa/Apa+ Apa/Api+ Api/Api−
Api09
P= 0
140
52
60
Pr(Apa/Apa)
Hybrid index
0.0
0.5
1.0

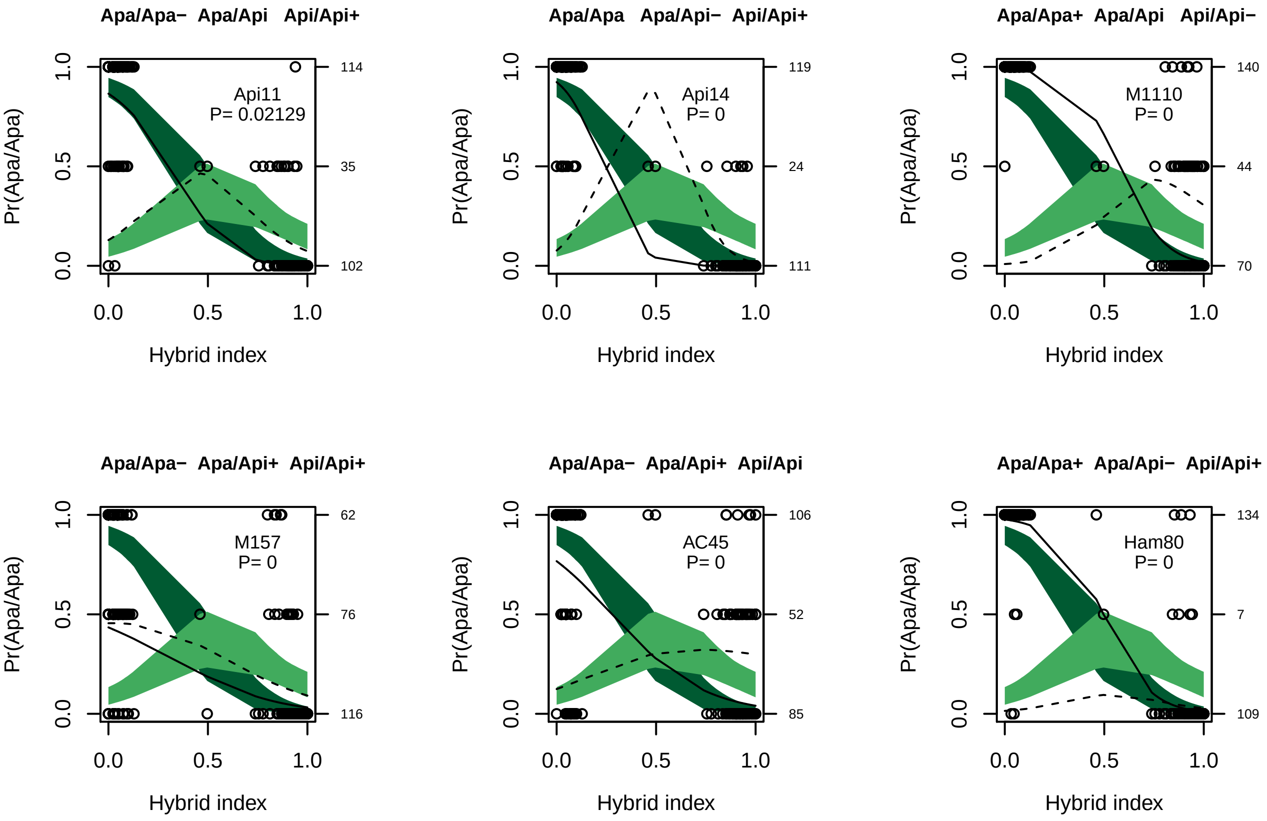

Apa/Apa− Apa/Api Api/Api+
Api11
P= 0.02129
114
35
102
Apa/Apa Apa/Api− Api/Api+
Api14
P= 0
119
24
111
Apa/Apa+ Apa/Api Api/Api−
M1110
P= 0
140
44
70
Apa/Apa− Apa/Api+ Api/Api+
M157
P= 0
62
76
116
Apa/Apa− Apa/Api+ Api/Api
AC45
P= 0
106
52
85
Apa/Apa+ Apa/Api− Api/Api+
Ham80
P= 0
134
7
109
Pr(Apa/Apa)
0.0
0.5
1.0
Hybrid index

Figure S2B

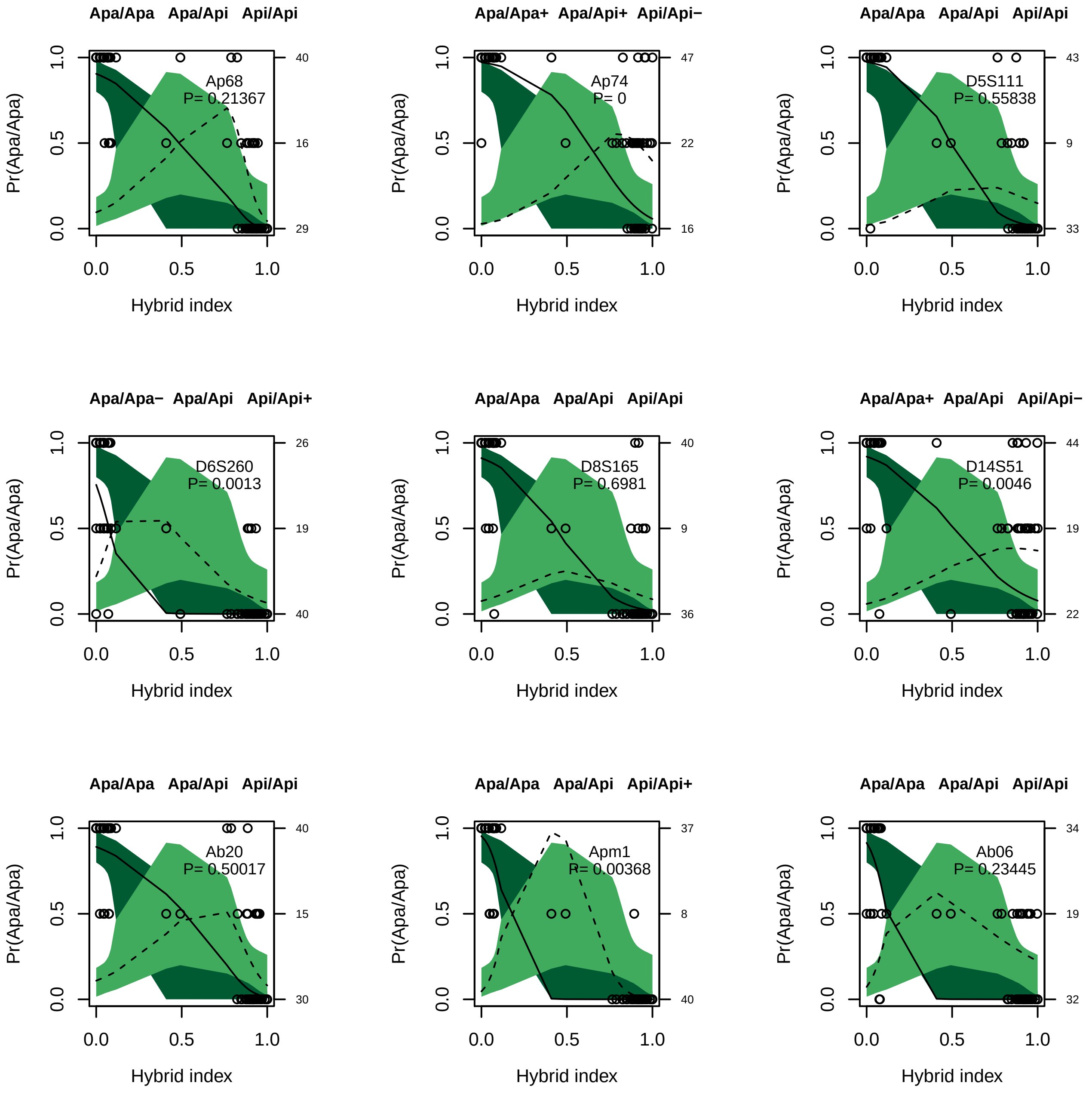

Apa/Apa Apa/Api Api/Api
Ap68
P= 0.21367
40
16
29
Apa/Apa+ Apa/Api+ Api/Api−
Ap74
P= 0
47
22
16
Apa/Apa Apa/Api Api/Api
D5S111
P= 0.55838
43
9
33
Apa/Apa− Apa/Api Api/Api+
D6S260
P= 0.0013
26
19
40
Apa/Apa Apa/Api Api/Api
D8S165
P= 0.6981
40
9
36
Apa/Apa+ Apa/Api Api/Api−
D14S51
P= 0.0046
44
19
22
Apa/Apa Apa/Api Api/Api
Ab20
P= 0.50017
40
15
30
Apa/Apa Apa/Api Api/Api+
Apm1
P= 0.00368
37
8
40
Apa/Apa Apa/Api Api/Api
Ab06
P= 0.23445
34
19
32
Pr(Apa/Apa)
Hybrid index
0.0
0.5
1.0

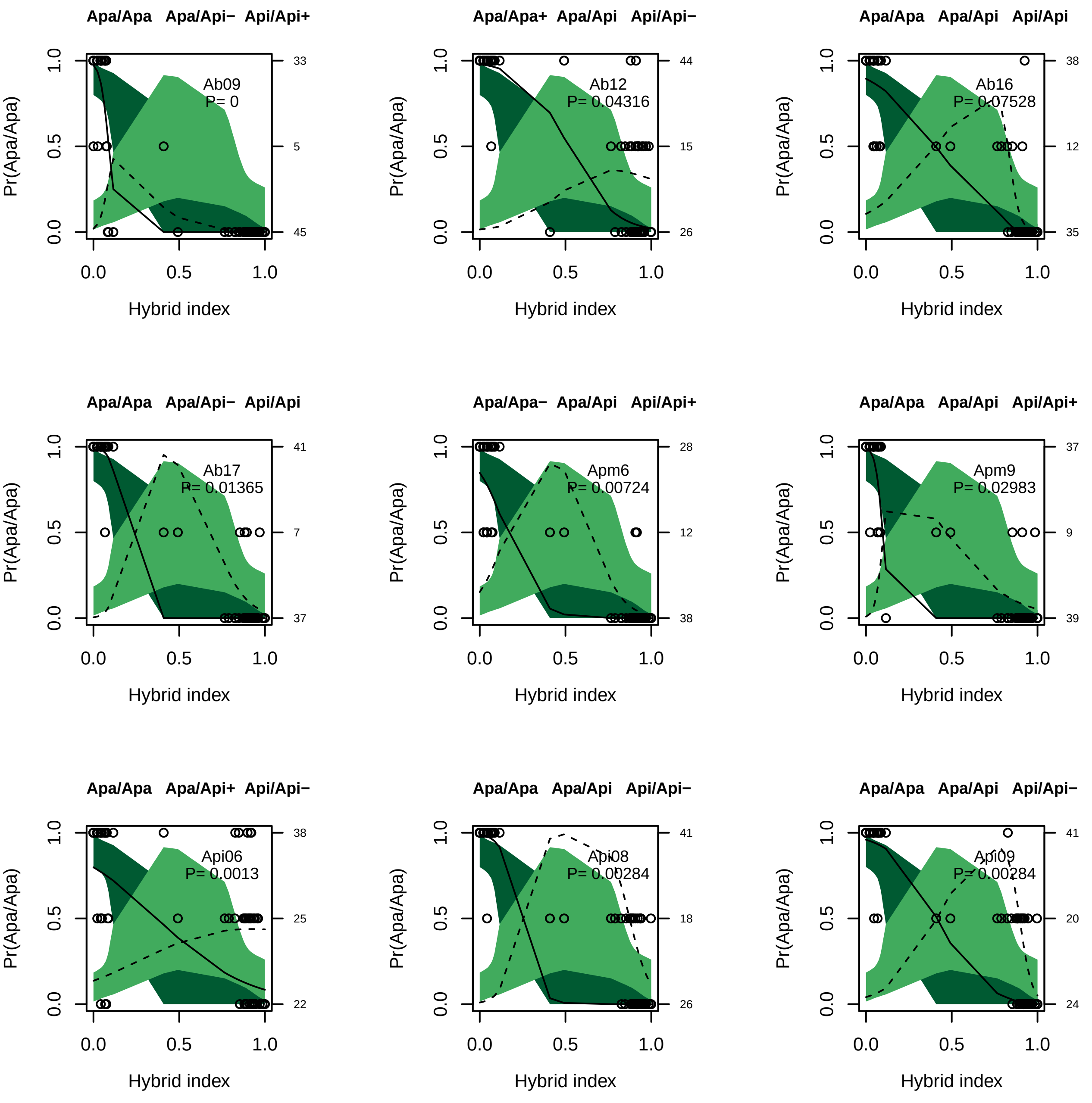
Apa/Apa Apa/Api− Api/Api+
Ab09
P= 0
Apa/Apa+ Apa/Api Api/Api−
Ab12
P= 0.04316
Apa/Apa Apa/Api Api/Api
Ab16
P= 0.07528
Apa/Apa Apa/Api− Api/Api
Ab17
P= 0.01365
Apa/Apa− Apa/Api Api/Api+
Apm6
P= 0.00724
Apa/Apa Apa/Api Api/Api+
Apm9
P= 0.02983
Apa/Apa Apa/Api+ Api/Api−
Api06
P= 0.0013
Apa/Apa Apa/Api Api/Api−
Api08
P= 0.00284
Apa/Apa Apa/Api Api/Api−
Api09
P= 0.00284
Pr(Apa/Apa)
Hybrid index

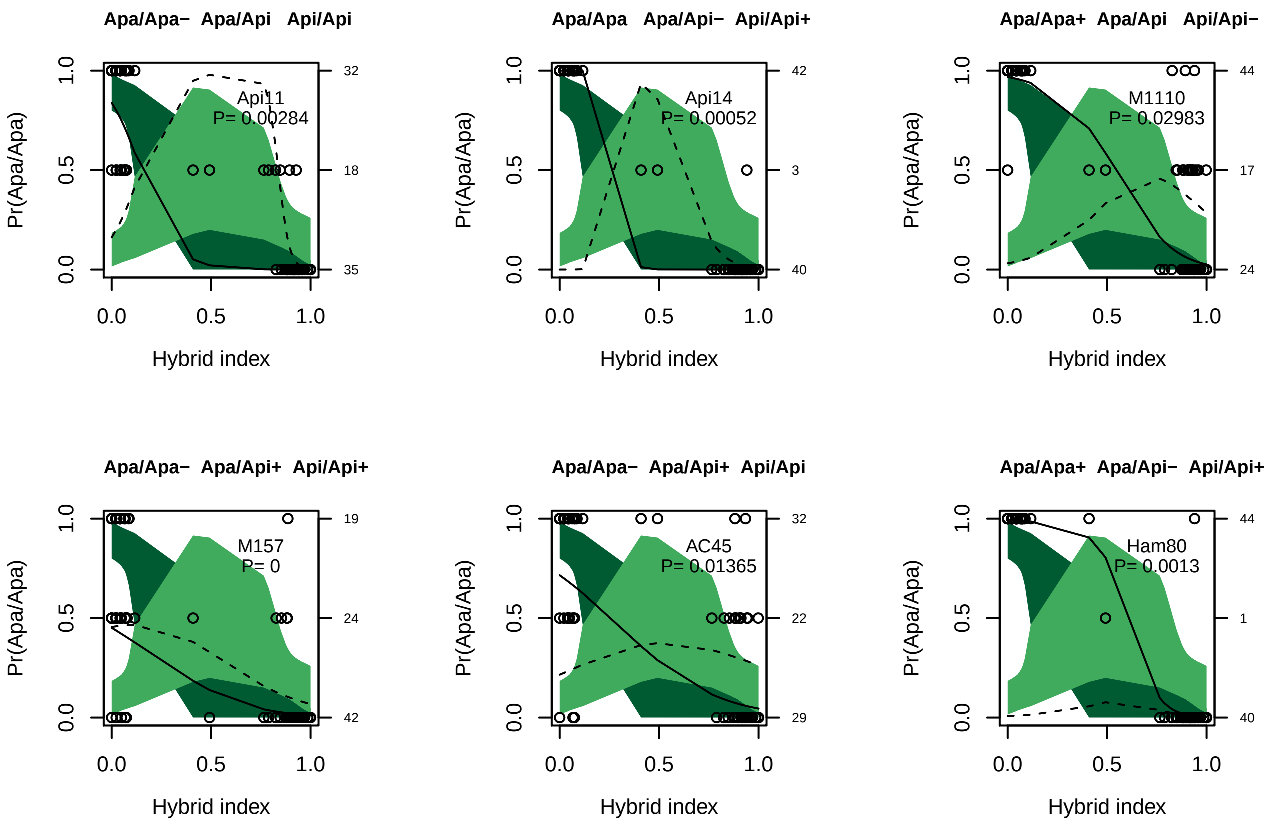

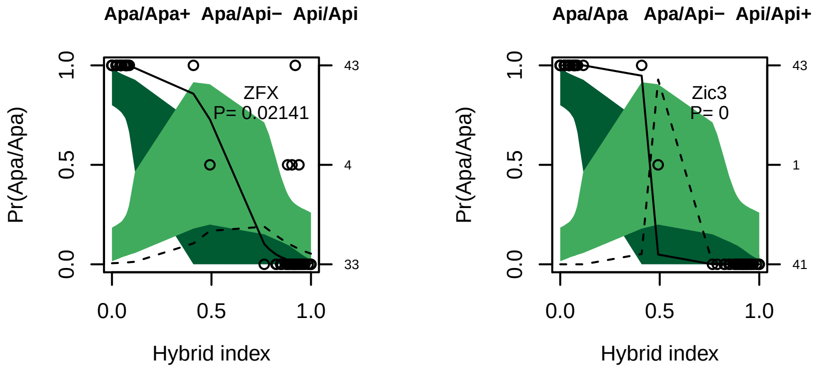

Fig. S2 Individual genomic clines for **(A)** all microsatellite loci based on the full dataset, and **(B)** all microsatellite and X-chromosome loci based on the reduced dataset.

Figure S3

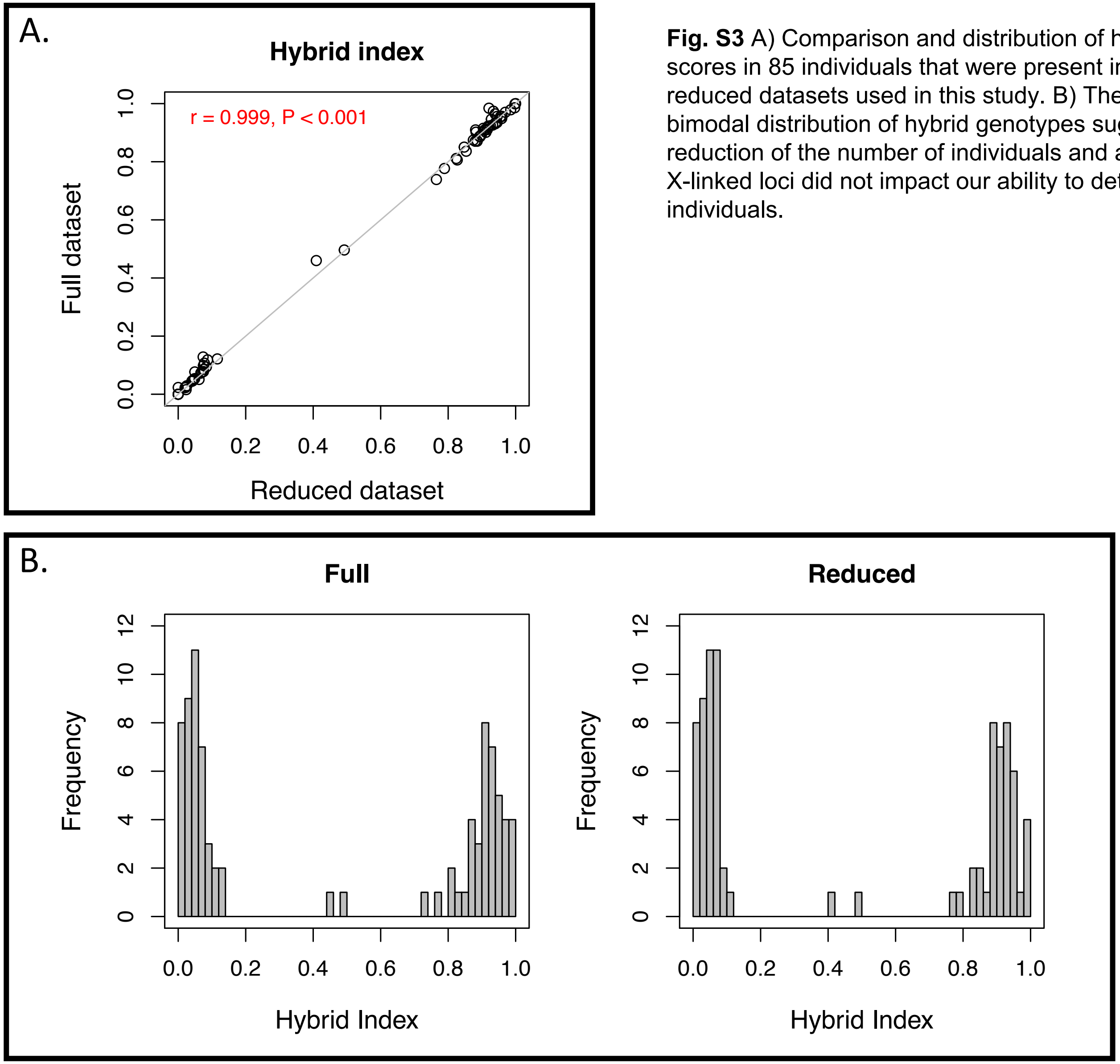

Fig. S3 A) Comparison and distribution of hybrid index (HI) scores in 85 individuals that were present in both full and reduced datasets used in this study. B) The similar bimodal distribution of hybrid genotypes suggests that the reduction of the number of individuals and addition of two X-linked loci did not impact our ability to detect admixed individuals.
