# Supplementary material for: Reduced Introgression of Sex Chromosome Markers in the Mexican Howler Monkey (Alouatta palliata × A. pigra) Hybrid Zone
Source: Int J Primatol. 2018 Sep 14;40(1):114–31. doi: 10.1007/s10764-018-0056-4 (PMC6394575; doi:10.1007/s10764-018-0056-4)
Supplement: Supplementary file 2 — (PDF 180 KB) [file 10764_2018_56_MOESM2_ESM.pdf]

Electronic Supplementary Material

Reduced Introgression of Sex Chromosome Markers in the Mexican Howler Monkey (*Alouatta palliata* × *A. pigra*) Hybrid Zone

Liliana Cortés-Ortiz · Marcella D. Baiz ·Javier Hermida-Lagunes · Francisco García-Orduña · Ariadna Rangel-Negrín · Dawn M. Kitchen · Thore J. Bergman · Pedro A. D. Dias · Domingo Canales-Espinosa

Table SI List of groups and individuals analyzed in this study. Because howler monkeys are heavily captured for pet trade in Mexico we refrain from disclosing the exact locations of the groups studied here. The coordinate data of our sampled groups are available from the corresponding author on reasonable request.

Group ID	Latitude	Longitude	Phenotype of individuals in groups	General locality	In the hybrid zone?	No. of individuals per group used in analyses
A	17.9958	–95.1756	*A. palliata*	Ver	N	5 (1M, 4F)
D	17.9886	–95.1992	*A. palliata*	Ver	N	6 (2M, 4F)
I	17.9886	–95.1992	*A. palliata*	Ver	N	2 (2M)
J	17.9958	–95.1756	*A. palliata*	Ver	N	2 (1M, 1F)
K	17.9958	–95.1756	*A. palliata*	Ver	N	2 (2M)
R	17.6425	–95.1378	*A. palliata*	Ver	N	5 (2M, 3F)
Y	17.9958	–95.1756	*A. palliata*	Ver	N	6 (2M, 4F)
78	18.4405	–95.0495	*A. palliata*	Ver	N	5 (3M, 2F)
79	18.0184	–95.1183	*A. palliata*	Ver	N	6 (5M, 1F)
L	18.8629	–90.7691	*A. pigra*	Camp	N	3 (2M, 1F)
M	19.3333	–90.6333	*A. pigra*	Camp	N	3 (2M, 1F)
U	17.9916	–88.8339	*A. pigra*	Q. Roo	N	4 (2M, 2F)
V	19.0530	–88.4311	*A. pigra*	Q. Roo	N	4 (2M, 2F)
W	20.6422	–87.5872	*A. pigra*	Q. Roo	N	4 (2M, 2F)
1	18.6954	–90.8746	*A. pigra*	Camp	N	8 (4M, 4F)
2	18.6707	–90.8379	*A. pigra*	Camp	N	4 (2M, 2F)
3	18.7047	–90.8859	*A. pigra*	Camp	N	4 (3M, 1F)
4	18.7511	–90.9586	*A. pigra*	Camp	N	8 (3M, 5F)
5	18.7177	–90.9024	*A. pigra*	Camp	N	7 (4M, 3F)
6	18.8101	–90.9811	*A. pigra*	Camp	N	3 (2M, 1F)
10	18.8962	–90.8870	*A. pigra*	Camp	N	7 (2M, 5F)
10A	18.8629	–90.7691	*A. pigra*	Camp	N	4 (2M, 2F)

11	18.7885	–90.9773	*A. pigra*	Camp	N	5 (2M, 3F)
12	18.7623	–90.9650	*A. pigra*	Camp	N	4 (1M, 3F)
13	18.8109	–90.9830	*A. pigra*	Camp	N	4 (2M, 2F)
20A	18.5703	–90.4383	*A. pigra*	Camp	N	4 (2M, 2F)
33	18.8085	–90.9779	*A. pigra*	Camp	N	4 (2M, 2F)
61	16.6349	–89.6532	*A. pigra*	Guatemala	N	8 (3M, 5F)
65	18.6093	–90.8103	*A. pigra*	Camp	N	6 (4M, 2F)
66	18.6143	–90.8075	*A. pigra*	Camp	N	7 (5M, 2F)
67	18.6165	–90.8050	*A. pigra*	Camp	N	4 (1M, 3F)
B	17.8422	–92.9978	*A. palliata*	Tab	Y	5 (1M, 4F)
E	17.6752	–92.6507	*A. palliata*	Tab	Y	4 (1M, 3F)
F	17.6742	–92.6498	*A. palliata* and *A. pigra*	Tab	Y	3 (1M, 2F)
O	17.6639	–92.6528	*A. palliata*	Tab	Y	5 (1M, 4F)
14	17.6849	–92.6889	*A. palliata*	Tab	Y	12 (3M, 9F)
22	17.7174	–92.6520	*A. palliata*	Tab	Y	4 (2M, 2F)
24	17.6149	–92.9025	*A. palliata*	Tab	Y	2 (1M, 1F)
25	17.5953	–92.9083	*A. palliata*	Tab	Y	6 (2M, 4F)
26	17.5933	–92.8685	*A. palliata*	Tab	Y	4 (2M, 2F)
42	17.6464	–92.6739	*A. palliata*	Tab	Y	14 (3M,11F)
43	17.6455	–92.6773	*A. palliata*	Tab	Y	1 (1F)
45	17.6831	–92.6708	*A. palliata*	Tab	Y	7 (6M,1 F)
49	17.7039	–92.6968	*A. palliata*	Tab	Y	7 (2M, 5F)
52	17.7304	–92.5549	*A. palliata*	Tab	Y	4 (2M, 2F)
53	17.7253	–92.5538	*A. palliata*	Tab	Y	6 (2M, 4F)
69	17.6456	–92.6666	*A. palliata*	Tab	Y	5 (1M, 4F)
72	17.6365	–92.6670	*A. palliata*	Tab	Y	12 (4M, 8F)
74	17.6349	–92.6707	*A. palliata*	Tab	Y	22 (5M, 17F)
75	17.6678	–92.6542	*A. palliata* and *A. pigra*	Tab	Y	3 (1M, 2F)
77	17.6591	–92.6610	*A. palliata*	Tab	Y	3 (2M, 1F)
81	17.6466	–92.6782	*A. palliata*	Tab	Y	6 (1M, 5F)
C	17.7391	–92.1342	*A. pigra*	Tab	Y	6 (3M, 3F)
G	17.7186	–92.6584	*A. pigra* and *A. palliata*	Tab	Y	3 (1M, 2F)
H	17.7100	–92.6661	*A. pigra*	Tab	Y	3 (1M, 2F)
N	17.6634	–92.6503	*A. pigra* and *A. palliata*	Tab	Y	4 (1M, 3F)
P	17.6676	–92.6541	*A. pigra*	Tab	Y	1 (1M)
S	17.6933	–92.6400	*A. pigra*	Tab	Y	5 (2M, 3F)
T	17.7293	–92.5067	*A. pigra*	Tab	Y	4 (3M, 1F)
15	17.7064	–92.6259	*A. pigra*	Tab	Y	6 (3M, 3F)
16	17.6993	–92.6482	*A. pigra*	Tab	Y	5 (1M, 4F)
19	17.7281	–92.5064	*A. pigra*	Tab	Y	4 (2M, 2F)
20	17.7125	–92.5096	*A. pigra*	Tab	Y	3 (2M, 1F)

21	17.7188	–92.5058	*A. pigra*	Tab	Y	5 (3M, 2F)
23	17.7911	–92.2897	*A. pigra*	Tab	Y	3 (2M, 1F)
40	17.6597	–92.6419	*A. pigra*	Tab	Y	2 (1M, 1F)
41	17.6904	–92.6597	*A. pigra*	Tab	Y	8 (3M, 5F)
44	17.6634	–92.6502	*A. pigra*	Tab	Y	5 (3M, 2F)
46	17.6628	–92.6549	*A. pigra*	Tab	Y	12 (6M, 6F)
47	17.6373	–92.5704	*A. pigra*	Tab	Y	6 (2M, 4F)
48	17.6374	–92.5807	*A. pigra*	Tab	Y	3 (1M, 2F)
50	17.7335	–92.4931	*A. pigra*	Tab	Y	1 (1F)
51	17.6529	–92.6313	*A. pigra*	Tab	Y	4 (4F)
68	17.6468	–92.6665	*A. pigra*	Tab	Y	6 (3M, 3F)
70	17.6375	–92.6694	*A. pigra*	Tab	Y	6 (1M, 5F)
71	17.6360	–92.6689	*A. pigra*	Tab	Y	6 (3M, 3F)
73	17.6905	–92.6597	*A. pigra*	Tab	Y	4 (2M, 2F)
76	17.6671	–92.6617	*A. pigra*	Tab	Y	5 (2M, 3F)

Table SII Collection, exportation, and importation permits of samples used in this study

Collecting permits	SGPA/DGVS/10637/11 (2011–2012)<br>SGPA/DGVS/03293/10 (2010–2011)<br>CESA/mam/Exp.7739<br>SGPA/DGVS/03676/07 (2007–2008)<br>SGPA/DGVS/01273/06 (2006–2007)<br>SGPA/DGVS/06116 (2005–2006)<br>SGPA/DGVS/03042 (2003–2004)<br>DOO.02–0789 (2001–2002)<br>DOO 750-10070/98 (1998–1999)
Exportation permits	CITES: MX 58954 (2012)<br>CITES: 199/2010 (2010)<br>CITES: MX 44458 (2009)<br>CITES: MX 40392 (2008–2009)<br>CITES: SER/A-03-05<br>CITES: MX 20802 (2004)
Importation permits	CITES: 12US46480A/9 (2013)<br>CITES: 10US10640A/9 (2011)<br>CITES: 09US199659/9 (2009)<br>CITES: 08US178005/9 (2008–2009)<br>CITES: SIM/A-21-03 (2004)<br>CITES: SIM/A007-2001 (2001)

Table SIII PCR conditions for amplification of microsatellite loci, mitochondrial control region, X chromosome loci, and the *SRY* gene

	Locus	Marker type	PCR cycles		Taq type	Used in analyses	Source
1	Ap68	Autosomal microsatellite	94° 2 min<br>94° 45 s<br>**50°** 30 s<br>72° 45 s<br>72° 10 min	35 cycles	P-Taq	Yes	Ellsworth and Hoelzer (1998)
2	Ap74	Autosomal microsatellite	94° 2 min<br>94° 45 s<br>**52°** 30 s<br>72° 45 s<br>72° 10 min	35 cycles	P-Taq	Yes	Ellsworth and Hoelzer (1998)
3	D5S111	Autosomal microsatellite	94° 2 min<br>94° 45 s<br>**60°** 45 s<br>72° 1 min<br>72° 10 min	35 cycles	P-Taq	Yes	Research genetics (see Cortés-Ortiz *et al.* 2010)
4	D6S260	Autosomal microsatellite	94° 2 min<br>94° 45 s<br>**53°** 45 s<br>72° 1 min<br>72° 10 min	35 cycles	P-Taq	Yes	Research genetics (see Cortés-Ortiz *et al.* 2010)
5	D8S165	Autosomal microsatellite	94° 5 min<br>94° 30 s<br>**55°** 30 s<br>72° 1 min<br>72° 10 min	35 cycles	P-Taq	Yes	Research genetics (see Cortés-Ortiz *et al.* 2010)
6	D14S51	Autosomal microsatellite	94° 5 min<br>94° 45 s<br>**60°** 45 s<br>72° 1 min<br>72° 10 min	35 cycles	P-Taq	Yes	Research genetics (see Cortés-Ortiz *et al.* 2010)
7	D17S804	Autosomal microsatellite	94° 2 min<br>94° 45 s<br>**60°** 45 s<br>72° 1 min<br>72° 10 min	35 cycles	P-Taq	No	Research genetics (see Cortés-Ortiz *et al.* 2010)
8	PEPC8	Autosomal microsatellite	94° 2 min<br>94° 30 s	40 cycles	P-Taq	No	Escobar-Páramo (2000)

			**46°** 30 s<br>72° 30 s<br>72° 10 min			
9	Ab06	Autosomal microsatellite	94° 2 min<br>94° 30 s<br>**60°** 30 s<br>72° 45 s<br>72° 10 min<br>35 cycles	P-Taq	Yes	Goncalves *et al.* (2004)
10	Ab07	Autosomal microsatellite	94° 2 min<br>94° 30 s<br>**60°** 30 s<br>72° 45 s<br>72° 10 min<br>35 cycles	P-Taq	Yes	Goncalves *et al.* (2004)
11	Ab09	Autosomal microsatellite	94° 2 min<br>94° 45 s<br>**58°** 45 s<br>72° 45 s<br>72° 10 min<br>35 cycles	P-Taq	Yes	Goncalves *et al.* (2004)
12	Ab12	Autosomal microsatellite	94° 2 min<br>94° 30 s<br>**65°** 30 s<br>72° 45 s<br>72° 10 min<br>35 cycles	P-Taq	Yes	Goncalves *et al.* (2004)
13	Ab16	Autosomal microsatellite	94° 2 min<br>94° 30 s<br>**65°** 30 s<br>72° 45 s<br>72° 10 min<br>35 cycles	P-Taq	Yes	Goncalves *et al.* (2004)
14	Ab17	Autosomal microsatellite	94° 2 min<br>94° 30 s<br>**60°** 30 s<br>72° 45 s<br>72° 10 min<br>35 cycles	P-Taq	Yes	Goncalves *et al.* (2004)
15	Ab20	Autosomal microsatellite	94° 2 min<br>94° 30 s<br>**67°** 30 s<br>72° 1 min<br>72° 10 min<br>35 cycles	P-Taq	Yes	Goncalves *et al.* (2004)
16	Apm1	Autosomal microsatellite	94° 2 min<br>94° 30 s<br>**64°** 30 s<br>72° 45 s<br>72° 10 min<br>35 cycles	P-Taq	Yes	Cortés-Ortiz *et al.* (2010)

17	Apm4	Autosomal microsatellite	94° 2 min<br>94° 30 s<br>**65°** 30 s<br>72° 30 s<br>72° 10 min<br>(35 cycles: 94° 30 s – 72° 30 s)	P-Taq	No	Cortés-Ortiz *et al.* (2010)
18	Apm6	Autosomal microsatellite	94° 2 min<br>94° 30 s<br>**55°** 30 s<br>72° 45 s<br>72° 10 min<br>(35 cycles: 94° 30 s – 72° 45 s)	P-Taq	Yes	Cortés-Ortiz *et al.* (2010)
19	Apm9	Autosomal microsatellite	94° 2 min<br>94° 30 s<br>**55°** 30 s<br>72° 45 s<br>72° 10 min<br>(35 cycles: 94° 30 s – 72° 45 s)	P-Taq	Yes	Cortés-Ortiz *et al.* (2010)
20	Api06	Autosomal microsatellite	94° 2 min<br>94° 30 s<br>**55°** 30 s<br>72° 45 s<br>72° 10 min<br>(35 cycles: 94° 30 s – 72° 45 s)	P-Taq	Yes	Cortés-Ortiz *et al.* (2010)
21	Api07	Autosomal microsatellite	94° 2 min<br>94° 30 s<br>**50°** 30 s<br>72° 45 s<br>72° 10 min<br>(35 cycles: 94° 30 s – 72° 45 s)	P-Taq	No	Cortés-Ortiz *et al.* (2010)
22	Api08	Autosomal microsatellite	94° 2 min<br>94° 30 s<br>**55°** 30 s<br>72° 45 s<br>72° 10 min<br>(35 cycles: 94° 30 s – 72° 45 s)	P-Taq	Yes	Cortés-Ortiz *et al.* (2010)
23	Api09	Autosomal microsatellite	94° 2 min<br>94° 30 s<br>**60°** 30 s<br>72° 45 s<br>72° 10 min<br>(35 cycles: 94° 30 s – 72° 45 s)	Hi-Fi Taq	Yes	Cortés-Ortiz *et al.* (2010)
24	Api11	Autosomal microsatellite	94° 2 min<br>94° 30 s<br>**55°** 30 s<br>72° 45 s<br>72° 10 min<br>(35 cycles: 94° 30 s – 72° 45 s)	Hi-Fi Taq	Yes	Cortés-Ortiz *et al.* (2010)

25	Api14	Autosomal microsatellite	94° 2 min<br>94° 30 s, **55°** 30 s, 72° 45 s — 35 cycles<br>72° 10 min	Hi-Fi Taq	Yes	Cortés-Ortiz *et al.* (2010)
26	1110	Autosomal microsatellite	94° 2 min<br>94° 30 s, **54°** 30 s, 72° 45 s — 35 cycles<br>72° 10 min	P-Taq	Yes	Di Fiore and Fleischer (2004)
27	157	Autosomal microsatellite	94° 2 min<br>94° 30 s, **54°** 30 s, 72° 45 s — 35 cycles<br>72° 10 min	P-Taq	Yes	Di Fiore and Fleischer (2004)
28	AC45	Autosomal microsatellite	94° 2 min<br>94° 30 s, **64°** 30 s, 72° 45 s — 35 cycles<br>72° 10 min	P-Taq	Yes	Oklander *et al.* (2007)
29	Ham80	X-chromosome microsatellite	94° 2 min<br>94° 30 s, **53°** 30 s, 72° 45 s — 35 cycles<br>72° 10 min	P-Taq	Yes	Katoh *et al.* (2009)
30	Zic3	X-chromosome gene sequence	95° 10 min<br>95° 15 s, 60-52 °C 30 s (60°C, 58°C, 56°C, 54°C and 52°C), 72° 1 min — 10 cycles (2 cycles per annealing Temp)<br>95° 15 s, 50° 30 s, 72° 1 min — 25 cycles<br>72° 30 min	GoTaq	Yes	Perelman *et al.* (2011)

31	Zfx	X-chromosome gene sequence	95° 10 min<br>95° 15 s<br>60-52 °C 30 s (60°C, 58°C, 56°C, 54°C and 52°C)<br>72° 1 min — 10 cycles (2 cycles per annealing Temp)<br><br>95° 15 s<br>50° 30 s<br>72° 1 min — 25 cycles<br>72° 30 min	GoTaq	Yes	Perelman *et al.* (2011)
32	SRY	Y-chromosome gene sequence	94° 2 min<br>94° 30 s<br>**60**° 30 s<br>72° 45 s — 35 cycles<br>72° 10 min	GoTaq	Yes	Moreira *et al.* (2002)
33	d-loop	Mitochondrial gene sequence	94° 2 min<br>94° 30 s<br>51° 30 s<br>72° 45 s — 35 cycles<br>72° 10 min	GoTaq	Yes	Ascunce *et al.* (2003); Dunn *et al.* (2014)
